# LasR-deficient *Pseudomonas aeruginosa* variants increase airway epithelial mICAM-1 expression and enhance neutrophilic lung inflammation

**DOI:** 10.1371/journal.ppat.1009375

**Published:** 2021-03-10

**Authors:** Lisa C. Hennemann, Shantelle L. LaFayette, Julien K. Malet, Perrine Bortolotti, Tianxiao Yang, Geoffrey A. McKay, Daniel Houle, Danuta Radzioch, Simon Rousseau, Dao Nguyen

**Affiliations:** 1 Department of Microbiology and Immunology, McGill University, Montreal, Quebec, Canada; 2 Meakins-Christie Laboratories, Translational Research in Respiratory Diseases, Research Institute of the McGill University Health Centre, Montreal, Quebec, Canada; 3 Department of Medicine, McGill University, Montreal, Quebec, Canada; 4 Department of Human Genetics, Research Institute of the McGill University Health Centre, Infectious Diseases and Immunity in Global Health Program Montreal, Quebec, Canada; University of North Carolina at Chapel Hil, UNITED STATES

## Abstract

*Pseudomonas aeruginosa* causes chronic airway infections, a major determinant of lung inflammation and damage in cystic fibrosis (CF). Loss-of-function *lasR* mutants commonly arise during chronic CF infections, are associated with accelerated lung function decline in CF patients and induce exaggerated neutrophilic inflammation in model systems. In this study, we investigated how *lasR* mutants modulate airway epithelial membrane bound ICAM-1 (mICAM-1), a surface adhesion molecule, and determined its impact on neutrophilic inflammation *in vitro* and *in vivo*. We demonstrated that LasR-deficient strains induce increased mICAM-1 levels in airway epithelial cells compared to wild-type strains, an effect attributable to the loss of mICAM-1 degradation by LasR-regulated proteases and associated with enhanced neutrophil adhesion. In a subacute airway infection model, we also observed that *lasR* mutant*-*infected mice displayed greater airway epithelial ICAM-1 expression and increased neutrophilic pulmonary inflammation. Our findings provide new insights into the intricate interplay between *lasR* mutants, LasR-regulated proteases and airway epithelial ICAM-1 expression, and reveal a new mechanism involved in the exaggerated inflammatory response induced by *lasR* mutants.

## Author summary

Cystic fibrosis (CF) patients develop progressive lung disease characterized by chronic airway infections, commonly caused by the opportunistic pathogen *Pseudomonas aeruginosa*, and excessive non-resolving neutrophilic inflammation. Loss of function mutations of the *lasR* quorum sensing transcription regulator gene commonly arise during chronic *P*. *aeruginosa* infections and are associated with increased lung inflammation. In this study, we demonstrated that loss-of-function *lasR* mutants induced increased mICAM-1 levels on airway epithelial cells compared to wild-type *P*. *aeruginosa* strains in cell culture and in murine infection models. This effect was caused by the loss of ICAM-1 degradation by LasR-regulated secreted protease, facilitated neutrophil adhesion, and was associated with increased neutrophilic lung inflammation. Our study provides novel insights into the *P*. *aeruginosa*—airway epithelial–neutrophil interactions, and demonstrates how a common pathoadaptation of *P*. *aeruginosa* may drive lung disease progression by exacerbating inflammation.

## Introduction

Individuals with the genetic disease Cystic Fibrosis (CF) develop progressive lung disease characterized by chronic airway infections and exuberant neutrophilic inflammation. Mutations in the Cystic Fibrosis Transmembrane Conductance Regulator (CFTR) gene lead to impaired chloride secretion and poor mucociliary clearance, making CF patients prone to lung infections. Chronic airway infections are major drivers of persistent inflammation in CF lung disease [1]. The majority of adult CF patients are infected with *Pseudomonas aeruginosa* for decades, which is associated with increased morbidity and mortality [2–4]. Within the airways, *P*. *aeruginosa* interacts with airway epithelial cells (AEC) to induce expression of pro-inflammatory mediators, modulate inflammatory pathways and further exacerbate lung inflammation [5]. Adding to a hyper-inflammatory state associated with the intrinsic CFTR defects [6,7], neutrophil responses to *P*. *aeruginosa* in the CF lung are both ineffective and excessive, further contributing to lung damage [8,9].

During chronic infection, *P*. *aeruginosa* evolves and genetically adapts to the host lung environment [10]. *P*. *aeruginosa* isolates recovered from chronic “late” stage CF infections are genotypically and phenotypically distinct from those found in “early” stage infections, and commonly share phenotypes such as mucoidy, loss of motility or protease production [11,12]. A notable example of this convergent evolution is the loss of LasR function, typically attributable to mutations in *lasR*, the gene encoding the major quorum sensing transcriptional regulator in *P*. *aeruginosa* [13–17]. Loss of function *lasR* variants are found in at least one third of CF patients chronically infected with *P*. *aeruginosa* [15]. Quorum sensing is a bacterial communication system that allows the coordinated expression of hundreds of *P*. *aeruginosa* genes, including many that encode exoproducts and virulence factors [18–20].

Although loss of LasR function leads to the attenuation of bacterial virulence in models of acute infection [12,21,22], chronic infections with *lasR* mutants in CF patients have been associated with accelerated decline in lung function [15] and increased markers of inflammation [23]. To understand the impact of *P*. *aeruginosa lasR* mutants on host-pathogen interactions and on the inflammatory responses relevant to CF lung disease, our group previously demonstrated that *lasR* variants elicited an enhanced pro-inflammatory cytokine response in AEC due to a loss of cytokine degradation by LasR-regulated proteases, leading to greater neutrophil recruitment *in vitro*. *lasR* variants also caused exaggerated neutrophilic inflammation and immunopathology in a murine model of sub-acute airway infection [23].

Adhesion molecules, such as cell surface intercellular adhesion molecule 1 (ICAM-1), play an important role in lung inflammation [24]. Membrane bound ICAM-1, expressed on endothelial and epithelial cells, is a ligand to β2 integrins on leukocytes and is involved in neutrophil migration, adhesion, and other functions [25–28]. Although the role of epithelial ICAM-1 is less well established than endothelial ICAM-1, which functions as the major adhesion receptor for leukocyte rolling-adhesion and transendothelial migration [28], studies suggest that airway epithelial ICAM-1 likely plays an important role in lung inflammation. Epithelial ICAM-1 mediates neutrophil adhesion and retention in the respiratory compartment, and contributes to lung inflammation in the setting of pulmonary infection and endotoxin-induced injury [26,29–33]. In fact, epithelial cells express low levels of ICAM-1 unless infected or stimulated by inflammatory cytokines [34,35] or bacterial products including *P*. *aeruginosa* secreted products [31,36,37]. Hubeau *et al* have also reported that AEC in the human CF lung over-express ICAM-1 compared to non-CF tissues, and ICAM-1 surface epithelial expression is associated with spatially adjacent neutrophil accumulation [38].

Since airway epithelial ICAM-1 expression is upregulated in *P*. *aeruginosa* infection, is a feature of CF lung disease and mediates neutrophilic inflammation in the lung, this led us to investigate the effect of *lasR* mutants on membrane bound ICAM-1 (mICAM-1) in AEC and its contribution to neutrophilic inflammation *in vitro* and *in vivo*.

## Material and methods

### Ethics statement

All experiments using human neutrophils were approved by the Research Ethics Board of the McGill University Health Centre (protocol 14–169), with informed written consent obtained from all subjects. All animal experiments were carried out with approval from the Animal Care Committee of the RI MUHC (AUP #2015–7586).

### Bacterial strains, plasmids and growth conditions

All bacterial strains used in this study are described in detail in S1 Table. Primer sequences and plasmids are listed in S2 and S3 Tables respectively. To generate the constructs for inducible protease expression, the *lasA*, *aprA* and *prpL* coding sequences were amplified by PCR from the PAO1 genome using primers *lasA*-GWB5-RBS, *lasA*-GWB2, *aprA*-GWB5-RBS, *aprA*-GWB2, *prpL*-GWB5-RBS and *prpL*-GWB2 pairs respectively. The PCR products were recombined into pDONR221P5P2 using BP clonase II to generate the entry vectors pENTR-*lasA*, pENTR-*prpL* and pENTR-*aprA*. The constructed entry vectors pENTR-*lasA*, pENTR-*prpL* and pENTR-*aprA* were each recombined with the vector pJJH187 and the destination vector miniCTX2.1-Tc-GW using LR Clonase II Plus (Invitrogen) to generate the arabinose inducible constructs pEXP-*lasA*, pEXP-*prpL* and pEXP-*aprA*. The individual expression vectors were subsequently integrated into the genome of the Late strain as previously described [23]. Complemented strains were selected on LB agar containing 50 μg/ml tetracycline. Protease expression was induced with 1% (w/v) L-arabinose (ACROS Organics) added to the growth media.

### *P*. *aeruginosa* filtrate preparation

Bacteria were grown overnight in 5 mL LB medium (BD Difco), washed twice in sterile phosphate-buffered saline (PBS) and the cell pellets were resuspended at an OD_600_ of 0.05 in synthetic CF medium (SCFM), which was developed to resemble the nutrient composition in CF sputum [39]. 5 mL planktonic cultures were incubated at 37°C for 24h, with shaking at 250 rpm, then centrifuged for 5 min at 5000 rpm to pellet cells. The supernatants were filtered with 0.22 μm cellulose acetate filters (Fisher Scientific) to generate sterile cell-free filtrates. The remaining cell pellets were resuspended in PBS to measure the OD_600_ for estimation of the cell biomass. Filtrates were first normalized to the OD_600_ of the pellet by dilution in SCFM and then stored at -20°C until use. As control, we note that the normalization of each filtrate to the OD_600_ or total protein content of the pellet were equivalent. Each filtrate aliquot was discarded after one freeze-thaw cycle. Where indicated, filtrates were heat-inactivated for 10 min at 95°C.

### Airway epithelial cell culture conditions and stimulation with *P*. *aeruginosa* filtrates

Immortalized human airway epithelial cells (BEAS-2B) were cultured in cell culture-grade plates in DMEM (Wisent) supplemented with 10% heat-inactivated fetal bovine serum (FBS, Wisent), penicillin and streptomycin (Wisent) at 37°C with 5% CO_2_. Once 80–90% confluent, cells were seeded with 150,000 cells/well into 12- well tissue culture plates (Sarstedt) for flow cytometry experiments, or with 37,500 cells per well in 48-well tissue culture plates (Sarstedt) for neutrophil adhesion assays. Cells were seeded 48 h prior to stimulation with filtrates, and 24 h before stimulation, the culture medium was changed to starvation media (DMEM containing penicillin, streptomycin and 0.5% heat-inactivated FBS). BEAS-2Bs were stimulated with 30 μL *P*. *aeruginosa* filtrate (in 1 mL total volume) for flow cytometry experiments or 7.5 μL filtrate (in 250 μL total volume) for neutrophil adhesion assays for a duration of 24 h in fresh starvation media at 37°C with 5% CO_2_. Equal volumes of SCFM were used as a negative control and a final concentration of 20 ng/mL TNF-α (BioLegend) was used as a positive control for ICAM-1 induction and neutrophil binding.

### mICAM-1 measurement

Following stimulation with *P*. *aeruginosa* filtrates, the supernatants of BEAS-2B cultures were collected and 500 μL cold Accutase (STEMCELL Technologies) was added to each well and incubated at room temperature for 10 min to detach cells. The BEAS-2B cell suspensions were then added to their respective supernatants of the same well and centrifuged at 2000 rpm. The cells were resuspended in FACS buffer (PBS+1% heat-inactivated FBS), stained with 1:1000 Fixable Viability Dye eFluor 780 (eBioscience) for 25 min on ice, then with 1:50 FITC-conjugated anti-human ICAM-1 antibody or IgG_1_-FITC isotype control (R&D Systems) for 30 min on ice, followed by fixation with 0.5% PFA for 10 min at room temperature. Cells were analyzed with a LSR II flow cytometer (BD Biosciences) and results were analyzed using FlowJo (BD Biosciences). For our analysis, debris (low FSC-A/SSC-A), doublets (higher SSC-A than SSC-H) and dead cells (high eFluor 780) were excluded. For each condition, the results were calculated by subtracting the median fluorescence intensity (MFI) of the isotype control from the corresponding sample’s ICAM-1 MFI.

### Protease activity measurements of *P*. *aeruginosa* filtrates

Total protease activity in filtrates was measured using the Hide-Remazol Brilliant Blue assay as previously described [40]. Caseinolytic activity in filtrates was assessed by spotting 30 μL of filtrates on sterile 6mm paper disk placed on skim milk 1.5% agar plates as previously described [41] and the zone of clearance (diameter minus the 6mm filter) was measured after 16 h incubation at 37°C. Elastolytic activity in filtrates was measured by Elastin-Congo Red (ECR) assay, as previously described in detail [23].

### Recombinant human ICAM-1 (rhICAM-1) degradation assay

To measure the ICAM-1 degradation by *P*. *aeruginosa* secreted proteases *in vitro*, 25 μL of 10 μg/mL (250 ng) rhICAM-1 (Peprotech) was incubated with 5 μL *P*. *aeruginosa* filtrate or PBS (negative control) for 24 h at 37°C, shaking at 200 rpm. The samples were then diluted in 4X loading buffer containing DTT, incubated at 95°C for 5 min, then stored at -20°C until quantification of rhICAM-1 by Western Blotting. 20 μL of each rhICAM-1 degradation sample was loaded onto 4–20% Mini-PROTEAN gels (Bio-Rad), separated by SDS-PAGE and then transferred onto PVDF membranes. The blots were blocked in 5% (w/v) skim milk prior to incubation with polyclonal anti-rhICAM-1 antibody (500-P287, Peprotech) overnight at 4°C. The protein bands were detected with anti-rabbit IgG DyLight 800 4X PEG conjugated secondary antibody (Cell Signaling Technology), imaged with the Odyssey imaging system (Li-Cor Biosciences) and quantified using the Odyssey V3.0 software (Li-Cor Biosciences). Results are shown as % band density compared to the negative control (rhICAM-1 incubated with PBS control) on the respective blot. Full blots are provided in S1 Spreadsheet.

### Neutrophil adhesion assay

Primary human neutrophils were isolated from 5 mL whole blood collected from healthy volunteer donors using the EasySep Direct Human Neutrophil Isolation Kit (Stemcell) according to the manufacturer’s instructions in phenol red-free RPMI-1640 with 1% heat-inactivated donor serum, and included incubation in RBC lysis buffer (Stemcell) for 10 min. Neutrophils were stained with 1 μM calcein-AM for 30 min in the dark at room temperature, then washed twice with media and filtered through a 40 μm nylon cell strainer cap (Fisherbrand). BEAS-2B cells were first stimulated with 30 μL *P*. *aeruginosa* filtrates for 24h, and after removal of the filtrate-containing media, BEAS-2B cells were co-incubated with 4x10^5^ neutrophils in phenol red-free DMEM supplemented with 1% heat-inactivated donor serum for 2 h at 37°C with 5% CO_2_. Neutrophils were then removed and BEAS-2B cells were washed three times with media to remove non-adherent neutrophils. For control experiments, 4x10^5^ neutrophils were stained and co-incubated with AEC as described above or incubated in wells containing media without AEC for 2h, then washed as described above. Adherent neutrophils were counted in a blinded manner using three representative fields of view per well obtained by laser scanning confocal microscopy (10X objective, Zeiss LSM700). Statistical analysis was performed using the average number of neutrophils per field of view (1.64 mm^2^) for each individual well.

### Subacute murine *P*. *aeruginosa* airway infection

Mice were infected with *P*. *aeruginosa* embedded in agar beads to generate a subacute non-lethal airway infections, as previously described [23]. Briefly, bacterial suspensions were mixed at 1:1 (v/v) in 2% LB and 3% molten agar with continuous stirring into mineral oil to generate bacteria embedded agar beads. Sterile PBS LB agar beads were used as a negative control. Adult male C57BL/6 mice (6 to 9 weeks old, Charles River) were infected by non-surgical intratracheal injection of 50 μL bead suspension containing ~5*10^5^ CFU/mouse. The mice were sacrificed at 2 or 4 days post infection (p.i). After cardiac puncture, the lungs were perfused by PBS injection into the vena cava to remove blood leukocytes prior to harvest. Perfused lungs were then lavaged with 4 x 0.5 mL ice-cold PBS through an intra-tracheal catheter for collection of the bronchoalveolar fluid (BALF). To measure the pulmonary bacterial load, lungs were harvested, homogenized and serially diluted for viable CFU counts on LB agar plates.

### Immune cell counts in lung homogenates and bronchoalveolar fluid (BALF)

For lung homogenates, perfused and lavaged lungs were placed in RPMI, minced, digested with 150 U/mL collagenase (Sigma-Aldrich) for 1 h at 37°C, then homogenized through a 16G needle. Cell suspensions were then filtered through a 100 μm cell strainer (BD Biosciences) and red blood cells were lysed with 0.2% (w/v) NaCl. The remaining cells were washed, resuspended in RPMI and enumerated using a Z1 cell counter (Beckmann-Coulter). Single cell suspensions were stained with Fixable Viability Dye eFluor780 (1:1000, eBioscience), blocked with anti-murine CD16/CD32 (1:100, eBioscience) then surface stained with eFluor610-conjugated anti-murine CD45 (1:40, 30-F11, eBioscience), FITC-conjugated anti-mouse CD11c (1:200, N418, eBioscience) and eFluor710-conjugated anti-murine Ly6G (1:160, 1A8, eBioscience). Cells were fixed with Cytofix (BD) and analyzed by flow cytometry using a LSR II Fortessa X-20 (BD Biosciences) and FlowJo 10.0.7 software (BD Biosciences). Neutrophils were defined as single, live, CD45+, Ly6G high and CD11c- cells, and total neutrophil counts were calculated by multiplying the proportion of neutrophils by the total number of live cells.

For the BALF, cells were spun down at 1000 rpm for 10 min, resuspended in PBS and counted by hemocytometer. For immune cell counts, cells collected from the BALF were loaded onto Shandon Cytoslides (Thermo Fisher), air-dried, stained with Shandon Kwik-Diff (Thermo Fisher) and analyzed by light microscopy to obtain average neutrophil, monocyte/macrophage and lymphocyte counts.

### Lung histopathology and ICAM-1 immunofluorescence

Perfused and lavaged lungs were inflated, fixed overnight with 10% formalin phosphate solution and sectioned in 5 μM thick slices. For histopathology, paraffin-embedded lung sections were stained with H&E and images were acquired using an Olympus BX51 microscope fitted with an Olympus DP70 CCD camera. For immunofluorescence, lung sections were stained with an anti-mouse ICAM-1 primary antibody (1:1000, eBioscience, YN1/1.7.4) and OmniMap anti-rat HRP (Ventana). All slides were processed using a Ventana DISCOVERY ULTRA automated slide preparation system (Roche) at the same time. The lung sections were imaged by laser scanning confocal microscopy (20x objective, Zeiss LSM700) at Ex 488 nm/Em 518 nm to visualize the autofluorescence of the lung tissues, and Ex 542 nm/Em 568 nm to visualize the ICAM-1 signal. All lung sections were imaged using identical confocal microscopy settings. Seven representative fields of view containing at least one airway cross-section were randomly selected per lung and imaged in a blinded manner.

Image analyses to quantify airway epithelial ICAM-1 expression were performed in two independent manners. First, the airway epithelial ICAM-1 expression of each airway was scored by visual assessment from 1 (no signal) to 10 (very strong) in a blinded manner by two independent reviewers. Second, ICAM-1 fluorescence intensity was quantified within the airway epithelium based on a manually designated region of interest (ROI) using the Icy software (V2.0.3.0, Institut Pasteur) [42]. The total fluorescence intensity of each ROI above a set threshold (defined by ICAM-1 negative regions in PBS lungs) was measured and normalized to the ROI surface area (in pixels).

### *P*. *aeruginosa* colony morphology

Bacteria were grown on LB agar for 16h. The presence of a metallic sheen caused by the accumulation of 4-hydroxy-2-heptylquinoline, which has previously been linked to loss of LasR function [16], was assessed visually.

### *N*-3-oxo-dodecanoyl-homoserine lactone (3-oxo-C12-HSL) measurement

Production of the LasR autoinducer 3-oxo-C12-HSL was measured as previously described [43,44]. Briefly, bacterial strains were grown in LB medium + 50 mM MOPS for 16h. 3-oxo-C12-HSL levels were quantified using the bioassay strain E. coli DH5α expressing pJN105L and pSC11.

### Statistical analyses

All results are shown as mean ± SD or SEM (as indicated), unless otherwise stated in the figure legends. Statistical analyses between two groups were performed using an unpaired two-tailed t-test or Mann-Whitney test as appropriate. Analyses between three or more groups were performed using a one-way ANOVA and Sidak’s multiple comparisons test. Correlation between two measurements were estimated by linear regression and statistical analysis was conducted using Pearson’s correlation analysis. Proportions within two groups were compared by Chi-Square test. Statistical analyses were done in Graphpad Prism 8.3.0 (GraphPad Software).

## Results

### Stimulation with LasR-deficient variants induces higher mICAM-1 levels in AECs than wild-type *P*. *aeruginosa*

**Fig 1:**
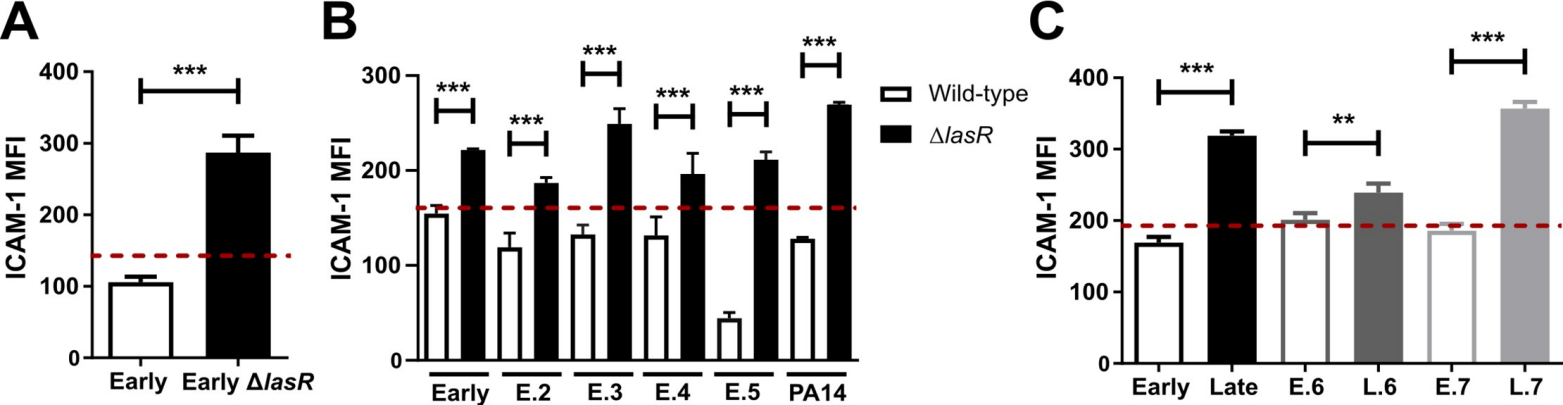
LasR deficient variants induce increased levels of mICAM-1 in AEC. BEAS-2B cells were stimulated for 24h with 30 μL sterile filtrates of (A) the Early clinical isolate and its isogenic *lasR* mutant, (B) five “early” clinical isolates or PA14 wild-type and their isogenic *lasR* mutants or (C) three “early” clinical isolates paired with their clonally-related “late” isolates (Early/Late, E6/L6, E7/L7). mICAM-1 levels (MFI) were measured by flow cytometry, with SCFM (media) serving as negative control (—dashed line). Results are shown as the mean ± SD of one representative experiment (from ≥ 2 independent experiments, each with biological triplicates). *P < 0.05; **P < 0.01; ***P < 0.001.

In order to compare the AEC mICAM-1 responses to secreted factors from loss-of-function *lasR* mutant and wild-type strains, we first stimulated BEAS-2B cells with filtrates of a *P*. *aeruginosa* clinical isolate isolated from early CF infection (“Early”) and its isogenic *lasR* mutant, and measured cell surface mICAM-1 levels by flow cytometry. We observed a 2.7-fold increase in mICAM-1 levels in cells stimulated with the *lasR* mutant filtrates compared to the wild-type filtrates (Fig 1A). Of note, stimulation with TNF-α was used as a positive control for mICAM-1 induction in every experiment (S1A Fig) and stimulation with SCFM medium, which was used as a negative control, did not induce significantly greater mICAM-1 expression than starvation media alone (S1B Fig).

To validate that the increased mICAM-1 response was attributable to the loss of LasR function in multiple *P*. *aeruginosa* strain backgrounds, we then stimulated AEC with filtrates from five other pairs of wild-type and isogenic *lasR* mutant strains, including four “early” clinical isolates (E.2 to E.5) and the common *P*. *aeruginosa* reference strain PA14. Stimulation with all *lasR* mutant filtrates induced mICAM-1 levels to a greater degree than their wild-type parental filtrates, ranging from a 1.4- to a 4.7-fold increase in mICAM-1 levels (Fig 1B). Furthermore, all mICAM-1 flow cytometry measurements were gated on live cells, and the cell viability ranged from 86.4% to 96.4% in different conditions (S1C Fig). We therefore conclude that these modest differences in cell viability were unlikely to be sufficient to explain the magnitude of mICAM-1 reduction observed in wild-type filtrate-stimulated AEC.

Since loss-of-function *lasR* mutations are commonly found in *P*. *aeruginosa* clinical isolates recovered during late stage CF infections, we next compared several “early” infection LasR-competent clinical isolates to their clonally related, “late” infection LasR-deficient isolates (characterized by low protease production, low 3-oxo-C12 HSL autoinducer levels and metallic colony sheen) [16] recovered from the same patients, namely the Early/Late, E.6/L.6 and E.7/L.7 paired isolates as characterized in S4 Table. We observed a similar pattern in mICAM-1 response, with stimulation by all “late” isolates eliciting a higher mICAM-1 response (Fig 1C). Both the Late and L.7 filtrate resulted in 1.9-fold greater mICAM-1 levels compared to their clonally related “early” isolates, but the difference in mICAM-1 levels was more modest (1.2-fold) with the E.6 - L.6 pair. The variability observed across the different “early-late” pairs was not surprising since “late” isolates harbour numerous genetic and phenotypic differences compared to their clonally related “early” isolates. Furthermore, different *lasR* mutations have varying effects on LasR function, and the LasR regulon can vary across different strain backgrounds [45]. These results thus indicated that loss-of-function *lasR* variants, both genetically engineered and naturally occurring ones, elicited higher mICAM-1 responses in human AEC than their respective wild-type counterparts, but that the magnitude of this phenotype varied depending on the *P*. *aeruginosa* strain background. Since the *P*. *aeruginosa* blue-green pigment pyocyanin can stimulate ICAM-1 expression [36] and is typically a LasR-controlled secreted secondary metabolite, we also noted that pyocyanin production was significantly decreased in the Early Δ*lasR* and Late strains compared to the Early strain (S1D Fig), thus indicating that the mICAM-1 expression in *lasR* mutant-stimulated AEC was unlikely to be attributable to increased pyocyanin levels.

### Increased mICAM-1 levels on *lasR* mutant-stimulated AEC correlate with decreased caseinolytic and elastolytic activity in *lasR* mutant filtrates

**Fig 2:**
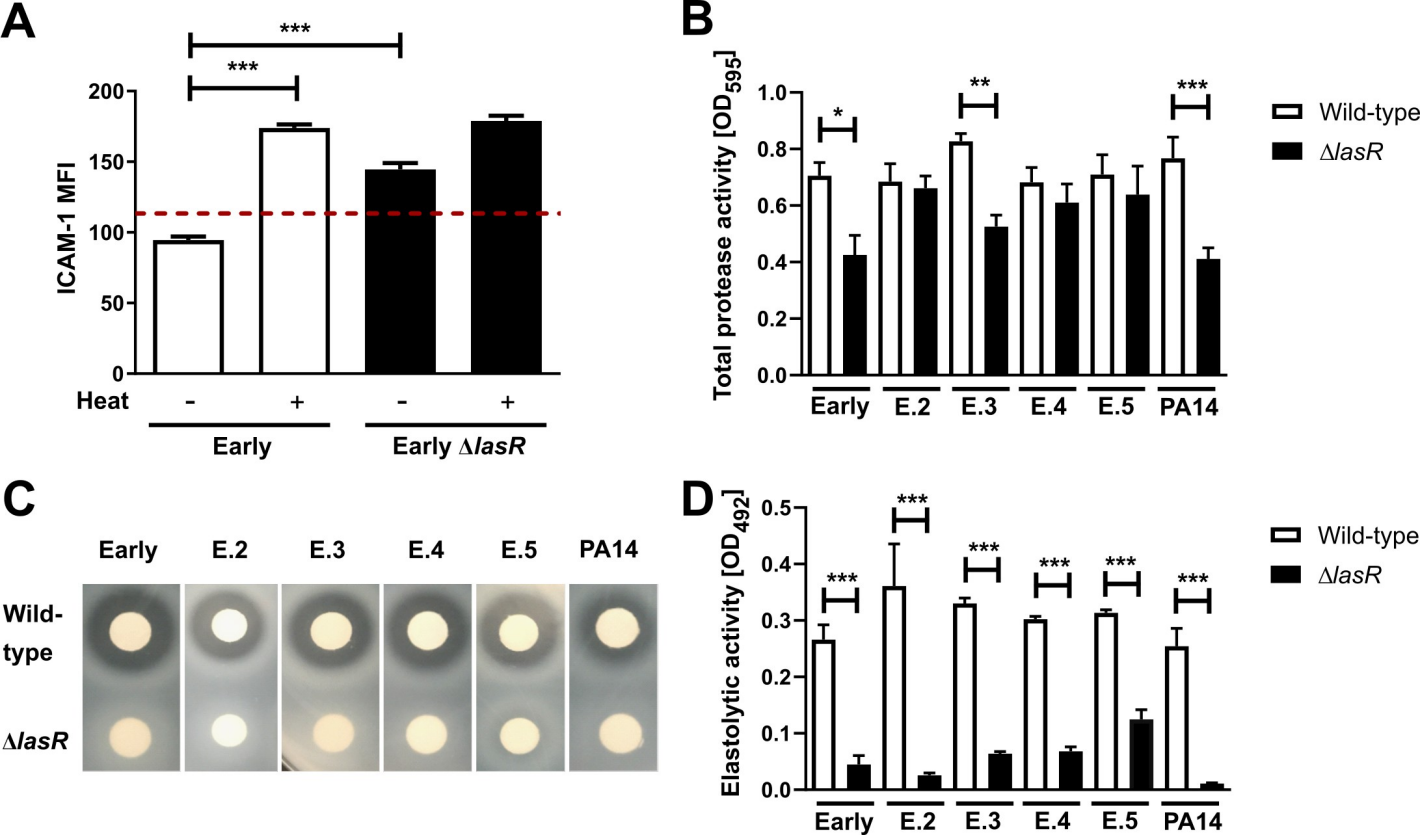
Induction of mICAM-1 is associated with reduced caseinolytic and elastolytic activity in *lasR* mutant filtrates. BEAS-2B cells were stimulated for 24h with 30 μL filtrates of (A) the Early and isogenic Early Δ*lasR* mutant (+/- heat inactivation). mICAM-1 levels (MFI) were measured by flow cytometry, with SCFM (media) serving as negative control (—dashed line). Protease activity in wild-type and *lasR* mutant filtrates was characterized for (B) total protease activity using the Hide-Remazol Brilliant Blue assay, (C) for caseinolytic activity using the clearance zone diameter on skim milk agar and (D) for elastolytic activity using the Elastin-Congo Red assay. Results for (A) are shown as mean ± SD of one representative experiment (from ≥ 2 independent experiments, each with biological triplicates). Results for (B) and (D) are shown as mean ± SEM, with pooled data (n ≥ 4 biological replicates from two independent experiments). Results for (C) are representative of ≥ 2 independent experiments in biological duplicates. *P < 0.05; **P < 0.01; ***P < 0.001.

In nearly all wild-type stimulation conditions, we also noted that mICAM-1 levels were below those observed in media control conditions (Figs 1A–1C and 2A), raising the possibility that mICAM-1 might be degraded or downregulated by wild-type filtrates. To start investigating this hypothesis, we heat-treated wild-type and *lasR* mutant filtrates prior to stimulation to inactivate heat labile bacterial exoproducts, which include secreted proteases. As shown in Fig 2A, heat inactivation of wild-type filtrates restored mICAM-1 to levels above those seen with non-heat treated *lasR* mutant filtrates, indicating that LasR-regulated heat-labile compound(s) present in wild-type filtrates degraded or down-regulated mICAM-1. We also noted that heat inactivation in both wild-type and *lasR* mutant filtrates led to a 1.8- and 1.2-fold increase in mICAM-1 levels compared to their respective non-heat-treated filtrates (Fig 2A), suggesting the concurrent presence of heat-stable compound(s) that either induced ICAM-1 production or prevented its degradation in both wild-type and *lasR* mutant filtrates.

LasR regulates the expression of several secreted proteases such as AprA, LasA, LasB and type IV protease (T4P) that can degrade components of host defenses and immunity [23,46–48], some of which have been shown to be heat-labile [23,49]. Therefore, we hypothesized that the loss of mICAM-1 protein signal upon stimulation with wild-type filtrates was due to the activity of these proteases, and sought to characterize the proteolytic activity in wild-type and *lasR* mutant filtrates. We measured protease activity in several manners, with a Hide Blue degradation assay for total protease activity, skim milk plates for caseinolytic activity and Elastin-Congo Red assay for elastolytic activity. Although the total protease activity only showed differences in three (Early, E.3, PA14) out of the six pairs (Fig 2B), caseinolytic (Fig 2C) and elastolytic activity (Fig 2D) were highly reduced (at least by 68%) or undetectable in all *lasR* mutants compared to parental wild-type strains. We also confirmed both caseinolytic and elastolytic activities to be heat-labile (S2A and S2B Fig). These results therefore indicated that there was a significant loss in caseinolytic and elastolytic activities among all *lasR* mutants tested. We further observed a negative correlation between the filtrates’ protease activity and ICAM-1 induction on AEC, with caseinolytic protease activity showing a stronger correlation (R^2^ = 0.76) than elastolytic protease activity (R^2^ = 0.66) (S2C and S2D Fig).

### LasR-regulated proteases degrade mICAM-1

**Fig 3:**
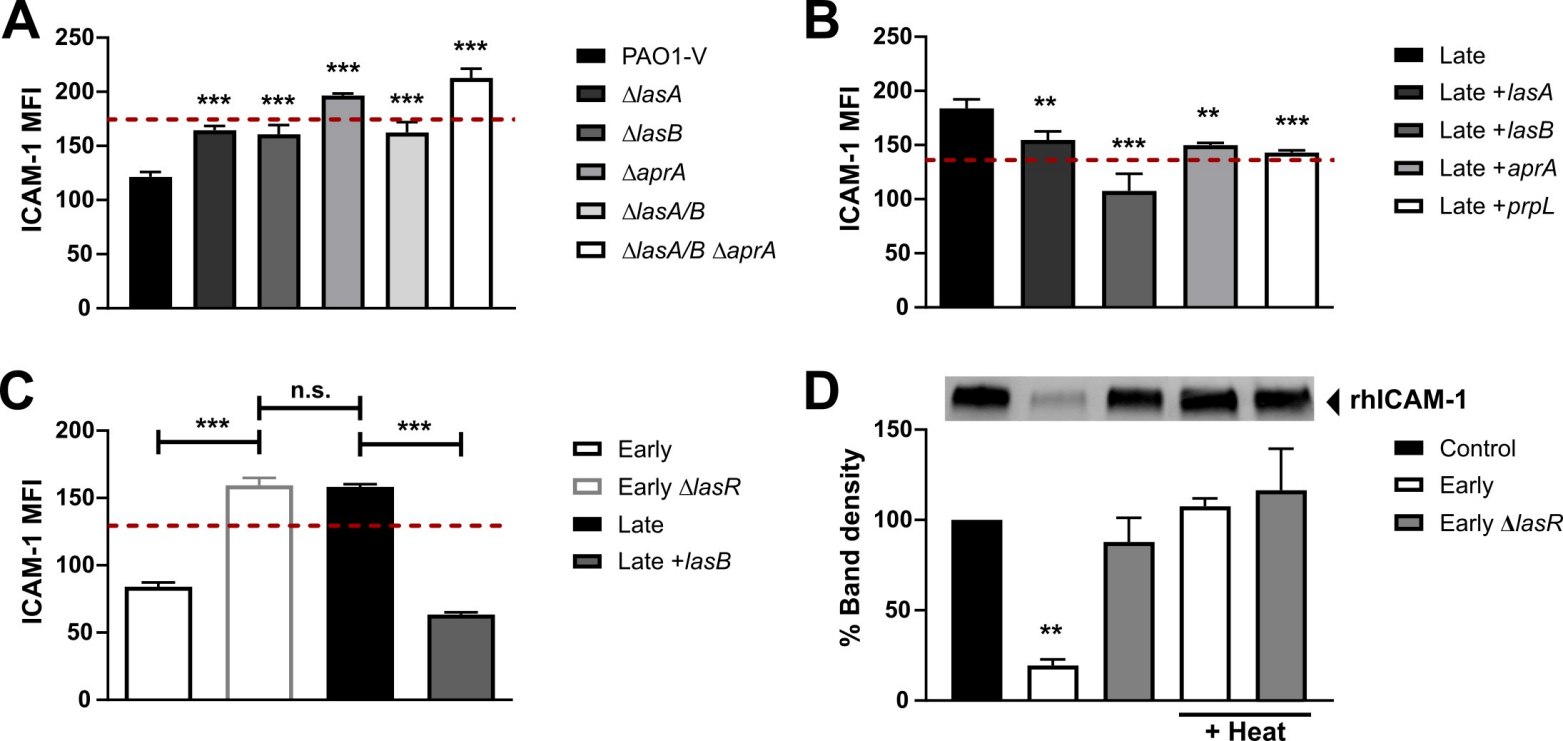
LasR-regulated proteases degrade ICAM-1. BEAS-2B cells were stimulated for 24h with 30 μL filtrates of (A) PAO1-V and its isogenic protease mutants of *lasA*, *lasB* and *aprA*, (B) the Late isolate and complemented strains Late +*lasA*, Late +*lasB*, Late +*aprA* or Late +*prpL* (T4P) or (C) Early, Early Δ*lasR*, Late, Late +*lasB*. mICAM-1 levels were measured by flow cytometry, with SCFM (media) serving as negative control (—dashed line). (D) in vitro degradation of recombinant human ICAM-1 (rhICAM-1) by *P*. *aeruginosa* filtrate. In each sample, 250 ng rhICAM-1 was incubated with 5 μL filtrates of the Early and Early Δ*lasR* strains (+/- heat inactivation) or PBS control for 24h, and the remaining intact rhICAM-1 following degradation was quantified by Western Blotting. Results in (A), (B) and (C) are shown as mean ± SD from one representative experiment (from ≥ 2 independent experiments, each with biological triplicates). Results in (D) are displayed as a representative Western Blot and quantification of the % band density compared to the PBS control condition (n ≥ 3 biological replicates from two independent experiments). Different lanes were cropped from the same blot and imaged at the same exposure. Full blots can be found in the S1 Spreadsheet. *P < 0.05; **P < 0.01; ***P < 0.001.

To test the contribution of the different LasR-regulated proteases on mICAM-1, we measured the effect of PAO1-V, an invasive *P*. *aeruginosa* isolate, and its single (*lasA*, *lasB*, *aprA*), double (*lasA/lasB*) and triple (*lasA/lasB/aprA*) protease mutants on mICAM-1 levels by flow cytometry. We observed a modest increase (1.3- to 1.4-fold) in mICAM-1 levels upon stimulation with filtrates from the Δ*lasA*, Δ*lasB* and Δ*lasA* Δ*lasB* strains (compared to wild-type) as well as a more pronounced increase (1.6- and 1.8-fold) upon stimulation with the Δ*aprA* and triple mutant, respectively (Fig 3A), suggesting that AprA might have the greatest effect on ICAM-1 levels in this strain background. The caseinolytic activity of the protease mutants further confirmed the relative contribution of each proteases in the PAO1-V strain background, as loss of AprA caused the greatest reduction in caseinolytic activity among the three proteases, while loss of all three LasB/LasB/AprA completely abrogated all caseinolytic activity (S3A Fig). Not surprisingly, elastolytic activity was nearly undetectable in the *lasB* and triple protease mutant (S3B Fig).

We further investigated the contribution of individual proteases by genetically complementing the Late (LasR-deficient) strain with the *lasA*, *lasB*, *aprA* or *prpL* (T4P) genes under control of an arabinose-inducible promoter to generate the Late +*lasA*, Late +*lasB*, Late +*aprA* and Late +*prpL* constructs (S1 Table). We confirmed that caseinolytic activity was increased upon complementation with the +*lasB*, +*aprA* and +*prpL* constructs (S3C Fig) and that elastolytic activity, which is mostly attributed to LasB, was only restored upon complementation with the +*lasB* construct (S3D Fig). Complementation with any of the four proteases resulted in decreased mICAM-1 levels compared to stimulation with the parental Late strain filtrate, with the greatest reduction in mICAM-1 levels (1.7-fold) observed with the Late +*lasB* strain (Fig 3B). Notably, *lasB* complementation of the Late isolate was sufficient to restore mICAM-1 levels to those observed in AECs stimulated with wild-type filtrates (Fig 3C).

To examine whether the decreased ICAM-1 levels induced by wild-type filtrates were due to direct degradation of ICAM-1, we incubated recombinant human ICAM-1 (rhICAM-1) with Early and Early Δ*lasR* filtrates and measured the *in vitro* rhICAM-1 degradation by Western blotting. Incubation with the Early, but not the Early Δ*lasR* filtrate, resulted in a significant reduction (81% decrease) in detectable and thus intact rhICAM compared to the PBS control (Fig 3D). We also confirmed that *in vitro* rhICAM-1 degradation was abrogated upon heat-inactivation of filtrates (Fig 3D), and reduced upon loss of one or more LasR-regulated proteases (S3E Fig). These results suggested that several LasR-regulated proteases present in wild-type filtrates can degrade rhICAM-1, the individual contribution of which might be strain-dependent.

### Neutrophil binding to AEC is increased upon stimulation with *lasR* mutant filtrates

**Fig 4:**
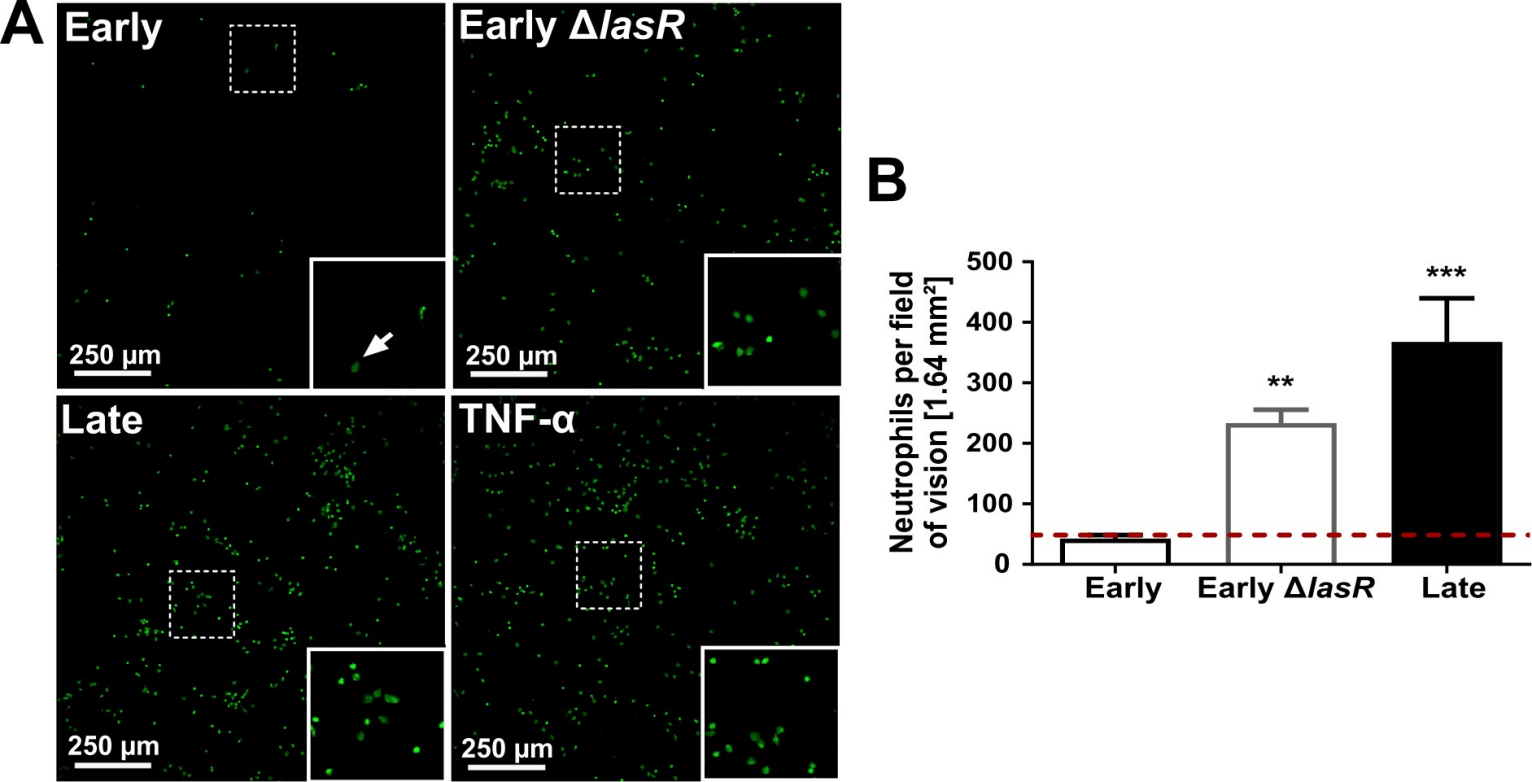
Neutrophil adhesion is increased in AEC stimulated with *lasR* mutant filtrates. BEAS-2B cells were pre-stimulated with 30 μL filtrates of Early, Early Δ*lasR* and Late strains, then co-incubated with calcein-stained primary neutrophils (green). 20 ng/mL TNF-α served as positive control and SCFM served as negative control (—dashed line). Adherent neutrophils were visualized by confocal microscopy (10X objective) in (A) and quantified in (B). Results are shown as mean ± SD from one representative experiment (from 2 independent experiments, each with 3 biological replicates). *P < 0.05; **P < 0.01; ***P < 0.001.

Next, we sought to characterize the functional consequences of the increased mICAM-1 responses in AEC stimulated with mutant *lasR* filtrates. Since mICAM-1 is involved in neutrophil adhesion to endothelial and epithelial cells [28,34,50], we hypothesized that AEC stimulated with *lasR* mutant filtrates will bind more neutrophils than AEC stimulated with wild-type filtrates. To test this, we quantified the adhesion of primary human neutrophils to AECs pre-stimulated with bacterial filtrates by confocal microscopy, and observed that significantly higher numbers of neutrophils adhered to AEC stimulated with *lasR* mutant filtrates (both Early Δ*lasR* and Late) compared to wild-type filtrate (5.5- and 8.6-fold increase respectively) or media control (Fig 4A and 4B). We also confirmed that neutrophil binding was specific to AEC, as control experiments using wells without AEC showed no adherent neutrophils (S3F Fig).

### The *lasR* mutant induces greater bronchial ICAM-1 levels and neutrophilic lung infiltration in a subacute murine lung infection model

We previously reported that *lasR* mutants induced a hyperinflammatory phenotype with higher levels of pro-inflammatory cytokines IL-6 and IL-8, greater neutrophilic inflammation and increased immunopathology compared to wild-type strains in a murine model of subacute lung infection [23]. In this well-established model, bacteria are embedded in agar beads and inoculated endotracheally, causing a non-lethal airway-centric infection that persist and is associated with neutrophilic inflammation. Our observations of ICAM-1 modulation *in vitro* therefore led us to ask whether *lasR* mutant infections were also associated with increased airway ICAM-1 expression *in vivo*. We infected C57BL/6 mice with wild-type or *lasR* mutant bacteria and analyzed the airway epithelial ICAM-1 expression by immunofluorescence, with confocal microscopy imaging of airway cross section on whole lung thin sections (S4A Fig). We analyzed mice at 2 and 4 days post-infection (p.i) and confirmed that both infection groups harboured equivalent bacterial burden at all time points (S4C Fig).

**Fig 5:**
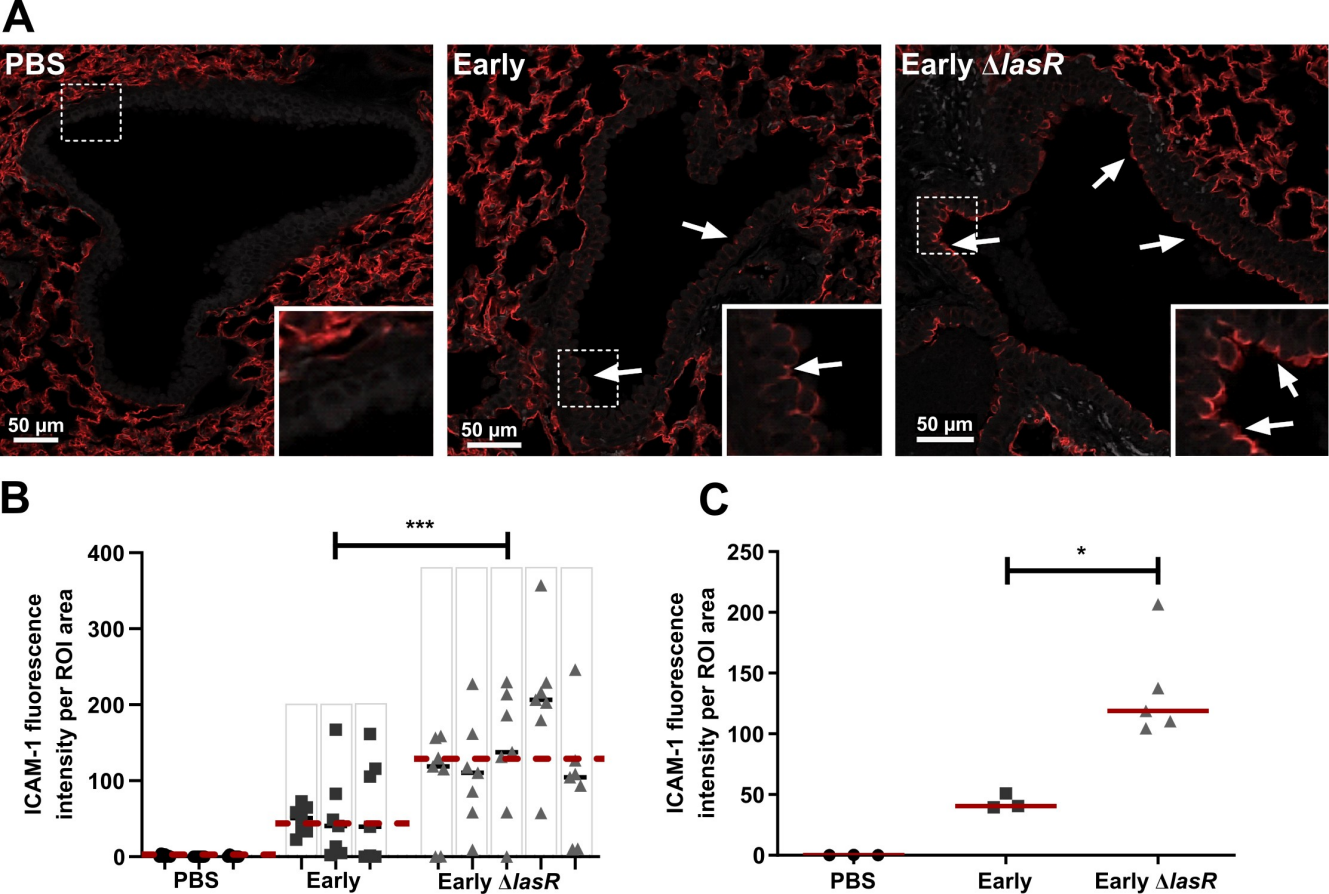
*lasR* mutant infected mice display increased airway epithelial ICAM-1 expression. Mice were infected with Early, Early Δ*lasR* or PBS embedded agar beads and lungs were harvested at 2 days p.i. for ICAM-1 immunofluorescence (shown as red). (A) Images shown are representative of airway cross-sections displaying the median ICAM fluorescence scores of each infection group. Images were taken with a 20X objective, inset boxes are digital magnification of the bronchial epithelium and the white arrows point to regions of high ICAM-1 expression localized to the apical epithelial surface (luminal side). The green autofluorescence (Ex 488/ Em 518, shown as grey) was imaged to visualize the tissue structure. (B) ICAM-1 total fluorescence normalized to the airway surface area (ROI) in every image, with results shown as median within each lung (black line) and median within each group (—dashed line), each dot corresponding to one distinct airway, and columns corresponding to one distinct mouse lung. (C) Median normalized ICAM-1 fluorescence, with results shown as medians using data pooled from 7 randomly selected airways per lung, and each dot corresponding to one mouse (n ≥ 3 mice per condition). *P < 0.05; **P < 0.01; ***P < 0.001.

Although ICAM-1 can be expressed by multiple cell types in the lung, including endothelial and alveolar epithelial cells, we focused on the ICAM-1 expression of the bronchial epithelium where we observed a strong induction in expression with *P*. *aeruginosa* infection, whereas ICAM-1 expression in the alveolar compartment remained constant in all conditions and time points (Fig 5A, area surrounding airways). To quantify ICAM-1 expression, we first developed an automated image analysis method to measure ICAM-1 signals across multiple airway cross-sections per lung section, and to normalize the total ICAM-1 fluorescence of each airway epithelial cross- section to its surface area (as outlined in S4A Fig). As highlighted in Figs 5A and S4B, bronchial ICAM-1 expression was largely restricted to the apical (luminal) side of the bronchial epithelium. We validated our automated ICAM-1 quantification method using a conventional semi-quantitative scoring system, and given the very good correlation between the two methods (R^2^ = 0.84, S4D Fig), we proceeded with the automated approach. The median ICAM-1 expression of all airways was significantly (3.2-fold) higher in *lasR* mutant infected mice compared to wild-type infected mice (Fig 5B). Analysis of pooled lung sections demonstrated a 2.9-fold higher median ICAM-1 airway signal in *lasR* mutant infected mice compared to wild-type infected mice at 2 days p.i, and consistently low (or undetectable) levels of ICAM-1 in PBS control mice (Fig 5C).

We also noted considerable heterogeneity in airway mICAM-1 expression within the same mouse lung (each mouse with its individual airway data points is represented in columns, Fig 5B) in both infection groups. This anatomically heterogeneous pattern was not surprising given that we used an airway infection model where agar bead embedded bacteria are entrapped within airway lumens and cause foci of infection rather than a diffuse infection throughout the lung [51]. As a result, lung regions in closest proximity to bacteria-containing beads displayed the greatest host responses to infection while more distant regions remained relatively normal, a pattern observed in other studies using a similar infection model [26,51]. By 4 days p.i, the majority of airways showed little to no mICAM-1 expression, and no significant differences were detected in the two infection groups (S4E Fig). While the proportion of ICAM-1 negative airways (i.e. airways with ICAM-1 signal comparable to PBS control) was not significantly different across both infection groups at 2 days p.i. (81% vs 91%, p = 0.251), we observed a trend towards a higher percentage of ICAM-1-positive airways in *lasR* mutant-infected mice at 4 days p.i. compared to wild-type-infected mice (40% vs 18%, p = 0.057, S4F Fig). Together, these results thus suggested that *lasR* mutants were associated with increased airway ICAM-1 induction both *in vitro* and *in vivo*.

**Fig 6:**
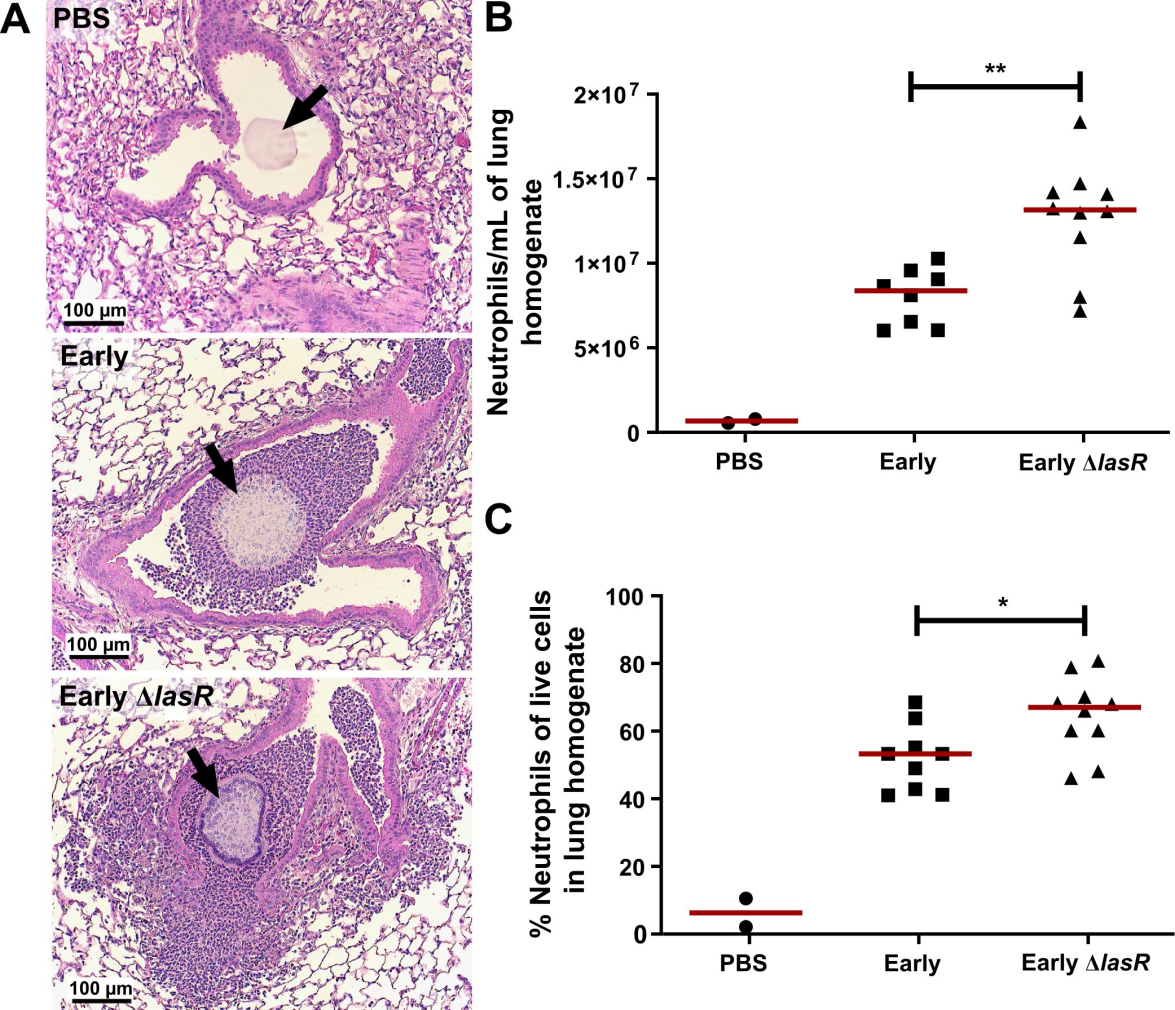
*lasR* mutant induces greater pulmonary neutrophilic inflammation than wild-type infection. Mice were infected with Early, Early Δ*lasR* or PBS embedded agar beads and lungs were harvested at 2 days p.i. for lung histopathology and immune cell counts. (A) Representative images of H&E stained lung sections (10X objective), demonstrating airway cross-sections and surrounding peri-bronchial tissues. Lung homogenates were analyzed by flow cytometry for enumeration of (B) total neutrophils (CD45+, Ly6G high, CD11c-) and (C) the percentage of neutrophils among all live single cells. Results are shown as medians, with each dot representing one mouse. *P < 0.05; **P < 0.01.

Next, we measured neutrophil counts in the lung homogenates and bronchoalveolar lavage fluid (BALF) to determine whether the increased ICAM-1 response to *lasR* mutant infections at 2 days p.i was also associated with increased lung neutrophilic infiltration. H&E staining of Early and Early Δ*lasR*-infected mouse lungs revealed that peri-bronchial and parenchymal inflammation in Early Δ*lasR*-infected mice was more extensive than in Early-infected or PBS control mice (Fig 6A). We note that the H&E lung sections could not be accurately assessed for intraluminal airway inflammation because the mouse airways were flushed with PBS for BALF collection prior to fixation. Both total lung neutrophil counts (1.6-fold increase, p = 0.006) and percentage of neutrophils (68% vs. 53%, p = 0.033) were significantly elevated in *lasR* mutant compared to wild-type infected mice (Fig 6B and 6C). We also noted no significant differences in the BALF analyses at day 2 p.i. (S5A and S5B Fig), although our previous studies using the same agar bead infection model showed that, by day 4 p.i, Early Δ*lasR*-infected mice displayed 25-fold greater BALF neutrophil counts compared to Early-infected mice (8.6x10^5^ vs 3.4x10^4^) [23]. Furthermore, our previous studies also reported greater BALF protein and more severe lung histopathology scores in Early Δ*lasR*-infected mice at day 4 p.i, confirming the presence of immunopathology in association with increased lung inflammation [23]. These results suggested that *lasR* mutants induced an early enhanced airway epithelial mICAM-1 response *in vivo* at 2 days p.i. which subsides by 4 days p.i. and are associated with increased neutrophil lung inflammation. Given that the bacterial burden in both Early and Early Δ*lasR* strains remained equivalent from the time of infection until day 4 p.i, the differences in ICAM-1 and inflammatory responses most likely resulted from differences in pathogen-host interactions rather than bacterial burden.

## Discussion

Loss-of-function *lasR* mutants are common in chronic CF infections [13,52], and are associated with more severe lung disease [15] and increased markers of inflammation [23] in CF patients. They cause dysregulated bacterial-host interactions through multiple mechanisms [23,46,53–55] that likely contribute to their propensity to cause greater pathology in chronic infection. Our lab has previously shown that *lasR* mutant infections caused an increased pro-inflammatory cytokine and chemokine response in airway epithelial cells, and an exaggerated neutrophilic inflammation and lung immunopathology *in vivo* [23]. In this study, we showed that loss-of-function *lasR* mutants also induced increased mICAM-1 levels on AEC compared to wild-type strains in cell culture models, and this effect facilitated neutrophil adhesion. We also found that *lasR* mutant infected mice showed increased airway epithelial ICAM-1 expression and neutrophilic lung inflammation compared to wild-type infected mice in a model of subacute airway infection. Our findings thus provide new insights into the intricate interplay between LasR-regulated proteases and airway ICAM-1 expression. As *P*. *aeruginosa* adapts to the CF lung and *lasR* variants emerge, these bacterial-host interactions and their effects in modulating lung inflammation change over the course of chronic infections.

Loss-of-function *lasR* mutations can emerge under laboratory conditions [16,56,57] and in human infections [58,59]. They are highly prevalent in chronic CF infections, as previously reported by our group and others [13,15,23,60], and numerous genomic studies of longitudinally collected *P*. *aeruginosa* clinical strains indicate that the *lasR* gene is under strong positive selection, with evidence of convergent evolution and pathoadaptation to the CF host [10,13,14,61–63]. Several studies have reported that loss-of-function *lasR* mutants have increased fitness in conditions such as low oxygen [64,65], denitrification [66], high cell density [67] and growth on certain amino acids [16,68]. It is therefore plausible that loss of LasR function confers a growth or survival advantage in such conditions relevant to the CF lung environment. We thus surmise that the exaggerated neutrophilic inflammation is a consequence rather than the selection pressure that drives the emergence of loss-of-function *lasR* variants in the CF lung.

Our results also demonstrated that the enhanced mICAM-1 response was primarily attributable to the loss of LasR-regulated proteases that degraded ICAM-1, findings which are consistent with previous reports by our group and others that LasR-regulated proteases can degrade mediators of the innate immune system and the complement system [23,46–48,54,69]. Furthermore, proteolytic cleavage of mICAM-1 by host-proteases such as neutrophil elastase and cathepsin G has been described [70,71], and degradation by bacterial proteases has been considered a potential mechanism of bacterial virulence [72]. While our previous findings suggested a key role for LasB in the degradation of IL-6 and IL-8 [23], we now observed that several LasR-regulated proteases, primarily LasB, AprA and T4P can degrade mICAM-1, and that their contributions likely vary depending on strain specific expression and secretion levels of each protease. The proteolytic activity of distinct proteases can also show interdependence, as LasA requires activation through proteolytic cleavage by LasB, and T4P function is significantly increased upon proteolytic cleavage by LasB and AprA [73,74].

*P*. *aeruginosa* can modulate ICAM-1 expression through other mechanisms. *P*. *aeruginosa* and other gram-negative bacterial lipopolysaccharide (LPS) can induce ICAM-1 expression in epithelial and other cell types [37,75,76]. Although LPS expression or structures may vary across *P*. *aeruginosa* clinical strains, we doubt that this variable had a major effect on the ICAM-1 induction by the Early and Early Δ*lasR* strains since heat-inactivated filtrates of both strains, which contain heat-stable compounds such as LPS, stimulated mICAM-1 to comparable levels (Fig 2A). The *P*. *aeruginosa* type III effector ExoU also induces cleavage of mICAM-1 to soluble ICAM-1, but through a host cyclooxygenase-dependent pathway [77,78]. We note that the effect of *P*. *aeruginosa* ExoU on mICAM-1 does not explain our results since ExoU secretion requires an active type III secretion system [79] and is likely negligible in the cell-free *P*. *aeruginosa* filtrates used in our *in vitro* experimental system. Finally, Look *et al*. have reported that phenazines (such as pyocyanin) secreted by *P*. *aeruginosa* also induce ICAM-1 expression in AEC [36]. However, these secondary metabolites are unlikely to account for the increased ICAM-1 response to our *lasR* mutant strains which are pyocyanin deficient (S1D Fig).

Our results support an important role for airway epithelial mICAM-1 responses in neutrophil adhesion. As an adhesion molecule expressed on the apical surface of epithelial cells [27,37], mICAM-1 allows immune cells, notably neutrophils, to bind to the airway epithelial surface, an interaction that promotes transepithelial migration and thus recruitment of inflammatory cells [26,30]. It also facilitates neutrophil-mediated clearance of pulmonary pathogens through yet unclear mechanisms [25,26]. We recognize that other adhesion molecules such as vascular cell adhesion molecule 1 (VCAM-1) also contribute to neutrophil adhesion to AEC. For example, VCAM-1 basal expression in AEC is low but upregulated in response to pro-inflammatory cytokine and respiratory syncytial virus [80–82]. This is consistent with our observation that neutrophil adhesion to AEC stimulated with Late filtrates is greater than Early Δ*lasR* filtrates despite similar mICAM-1 levels in both conditions, indicating that mICAM-1 is not the sole adhesion factor involved.

LasR regulates the expression of many secreted proteins and small molecules. We thus recognize that loss-of-function *lasR* variants likely modulate host inflammatory responses through additional pathways. For example, LasR-regulated secreted molecules such as pyocyanin and rhamnolipids can cause cell death, dampen immune cell function and trigger inflammation [83–85]. LasR-regulated proteases can degrade a broad range of host proteins that mediate lung inflammation, from cytokines such as IL-6, IL-8, MCP-1 and IFN-γ [23,48,69,86] to protease-activated receptors such as PAR2 [87,88], as well as flagellin monomers, a well established pathogen-associated molecular pattern [89,90]. Although the ability of LasR-regulated proteases to target host immune and inflammatory mediators has typically been considered as a mechanism of immune evasion in the context of acute infections, we propose that the loss of such activity contributes in fact to the hyper-inflammation and immunopathology observed in the setting of subacute or chronic infections.

In our *in vivo* infection model, we observed that *lasR* mutant infections were associated with higher pulmonary neutrophil counts by flow cytometry and peri-bronchial inflammation by immunohistopathology. While BALF neutrophil counts did not differ significantly at 2 days p.i., we have previously reported that they were significantly increased at 4 days p.i. in Early Δ*lasR*-infected mice [23]. This suggests that neutrophil recruitment into the airways likely lags and manifests later than in the peri-bronchial compartments. Although we cannot directly infer the causal contribution of airway ICAM-1 to the pulmonary neutrophilic inflammation in our *in vivo* model, the association between the two is consistent with our *in vitro* neutrophil adhesion data.

The role of airway epithelial mICAM-1 in lung inflammation during infection is emerging but complex and incompletely understood. Previous studies have also suggested that airway epithelial mICAM-1 expression significantly affects neutrophil recruitment to the lungs *in vivo* and *in vitro* [26,34]. For example, in a model of subacute *H*. *influenzae* lung infection, Humlicek *et al* demonstrated a marked reduction of leukocyte recruitment upon intratracheal instillation of anti-ICAM-1 blocking antibody which predominantly targets epithelial cells of the lungs [26]. Studies have reported that basal mICAM-1 expression is minimal in the airway epithelium in human and rodent lung tissues and cell cultures, but induced in states of inflammation and infection [29,38,82]. This stands in contrast to alveolar epithelial cells which show high constitutive expression levels of mICAM-1 in the absence of infection, as we and others observed in mice, rats and humans [33,91]. Induction of mICAM-1 expression in AEC has been observed with infection by *H*. *influenzae*, *C*. *pneumoniae*, *Pneumocystis carinii* [26,29–31] or *P*. *aeruginosa* [36] as seen in our study, either directly in response to bacterial products, or indirectly in response to inflammatory cytokines such as TNF-α [82,92]. Epithelial ICAM-1 thus promotes neutrophil recruitment and retention to the airway compartment [24,26], a response that may aid in pathogen clearance during infection but also exacerbates inflammation-mediated pathology. Our study thus demonstrates that loss-of-function *lasR* mutants induce greater mICAM-1 expression and neutrophil adhesion to human airway epithelial cells *in vitro*. We also observed that *lasR* mutant infections elicit greater ICAM-1 expression in the bronchial epithelium and greater lung inflammation in murine infections, but this association remains to be mechanistically proven *in vivo*.

In conclusion, we report on a novel mechanism through which *P*. *aeruginosa* modulates innate immune and inflammatory responses in the host lung, and how loss of LasR function, a common patho-adaptation during chronic CF infections, enhances neutrophilic inflammation. Additionally, modulation of airway epithelial mICAM-1 expression may also have other implications through its function as the major entry receptor of human rhinoviruses (HRV) [93]. For example, stimulation of AEC with *H*. *influenzae*, which also induces mICAM-1 expression, leads to increased susceptibility to HRV-infection [94]. Whether AEC stimulated with *P*. *aeruginosa lasR* mutants are more susceptible to HRV infection remains to be determined. HRV infections are common in CF patients and may be linked to pulmonary exacerbations [95], an important determinant of lung function decline in CF patients. Whether CF patients chronically infected with *P*. *aeruginosa lasR* mutants are more susceptible to HRV infection as a result of enhanced airway mICAM-1 levels is an intriguing hypothesis to be explored.

## Supporting information

S1 Fig Controls for mICAM-1 induction and AEC viability. BEAS-2B cells were stimulated for 24h with (A) 30 μL filtrate of the Early or Early Δ*lasR* strain or 20 ng/mL TNF-α; (B) starvation media (SM) +/- 30 μL SCFM medium; (C) 30 μL filtrates from six pairs of wild-type clinical isolates and isogenic *lasR* mutant. In (A) and (C), SCFM served as negative control (—dashed line) and 20 ng/mL TNF-α as positive control. (A+B) mICAM-1 levels were measured by flow cytometry and (B) AEC viability following filtrate stimulation was measured as the percentage of live cells (low eFluor 780) among all single cells by flow cytometry. The results are shown as mean ± SD of one representative experiment (from ≥ 2 independent experiments, each with biological triplicates). (D) Representative bacterial cultures of the Early, Early Δ*lasR* and Late strains, with pyocyanin (blue-green pigment) production only evident with the Early strain. Cultures were grown in SCFM, as used for filtrate production. *P < 0.05; **P < 0.01; ***P < 0.001.
(TIF)

S2 Fig Secreted protease activity in *P*. *aeruginosa* filtrates is heat-labile and negatively correlated with mICAM-1 levels in filtrate-stimulated AEC. (A) Caseinolytic activity in Early and Early Δ*lasR* filtrates (+/- heat treatment) was measured on skim milk agar plates. (B) Elastolytic activity in Early and Early Δ*lasR* filtrates (+/- heat treatment) was measured by Elastin-Congo Red assay. Correlation between (C) caseinolytic or (D) elastolytic activity of different *P*. *aeruginosa filtrates* and mICAM-1 levels on AEC stimulated with the respective filtrates. Results in (A) and (B) are shown as mean +SEM and are representative of ≥ 2 independent experiments, each with biological duplicates. In (C) and (D), each data point represents one wild-type or *lasR* mutant strain, with the X value displaying the mean ±SD ICAM-1 induction in one representative experiment (in biological triplicates) and the Y value displaying the mean ± SEM (C) caseinolytic or (D) elastolytic activity (two independent experiments, each with biological duplicates). The trendlines in (C) and (D) were calculated by linear regression. ND = not detectable. *P < 0.05; **P < 0.01; ***P < 0.001.
(TIF)

S3 Fig Controls for secreted protease mutants and complementation. Caseinolytic (A+C) and elastolytic (B+D) activity of (A+B) PAO1-V and its isogenic protease mutants of *lasA*, *lasB* and *aprA* or (C+D) the Late strain complemented with *lasA*, *lasB*, *aprA* or *prpL* (T4P) was measured on skim milk agar plates and by Elastin-Congo Red assay, respectively. (E) rhICAM-1 was quantified by Western Blotting with a polyclonal anti rhICAM-1 antibody, following incubation for 24h with PBS (- control) or filtrates of PAO1-V and its isogenic protease mutants as indicated. (F) Adhesion of calcein-stained human primary neutrophils (green) after 2h of incubation in wells with or without AEC was analyzed by confocal imaging. Results in (A-D) are shown as mean ± SEM, with pooled data (n ≥ 3 biological replicates from ≥ 2 independent experiments). Results in (G) are representative of 2 independent experiments. Results in (F) are representative of 2 independent experiments. *P < 0.05; **P < 0.01; ***P < 0.001.
(TIF)

S4 Fig ICAM-1 immunofluorescence and bacterial burden in mouse infection model. (A) Representative image of the region of interest (ROI, green area) manually drawn to define the airway epithelium on mouse lung sections. (B) Representative image of an airway cross-section (20X objective, with digital magnification in the inset box) displaying high bronchial ICAM-1 expression (red) localized to the apical side the bronchial epithelium facing the lumen (arrows). The autofluorescence of the tissue was imaged in the green channel (Ex 488/ Em 518) and is shown both in grey (to better highlight the ICAM-1 signal) or green. (C) Total lung bacterial burden at 2 and 4 days p.i, in mice infected with the Early or Early Δ*lasR* strain. Results are pooled from two independent experiments. (D) Correlation between manual and automated scoring of the airway epithelial mICAM-1 fluorescence intensity. Each dot represents one distinct airway section analyzed. (E) ICAM-1 fluorescence intensity per ROI area in mice infected with the Early, Early Δ*lasR* or PBS control. Each dot represents one airway section. (F) Percentage of ICAM-1 positive airways in mice infected with the Early or Early Δ*lasR* strain at 2 and 4 days p.i. Results are shown as mean ± SEM (C), median ± IQR (E) or percentages (F). *P < 0.05; **P < 0.01; ***P < 0.001; n.s. *P* ≥ 0.05.
(TIF)

S5 Fig Neutrophilic inflammation in mouse BALF. C57BL/6 mice were infected with the Early, Early Δ*lasR* or PBS control and sacrificed at 2 days p.i. (A) In BALF, the proportion of neutrophils was determined by Kwik-Diff staining. (B) Total BALF neutrophil counts, calculated by multiplying the proportion of neutrophils by the total number of live cells. Results are shown as medians (n = 2 mice in the control group, n ≥ 8 mice in infected groups). *P < 0.05; **P < 0.01; ***P < 0.001.
(TIF)

S1 Table Strains used in this study. (DOCX)

S2 Table Primers used in this study. (DOCX)

S3 Table Plasmids used in this study. (DOCX)

S4 Table Characteristics of paired clonally related early and late infection isolates. Low protease production, the presence of a metallic sheen and low 3-oxo-C12-HSL signal level are characteristic phenotypes of LasR-deficient strains. NA not available.
(DOCX)

S1 Spreadsheet Minimal dataset. (XLSX)
