# Supplementary figures and images for: LasR-deficient Pseudomonas aeruginosa variants increase airway epithelial mICAM-1 expression and enhance neutrophilic lung inflammation
Source: PLoS Pathog. 2021 Mar 10;17(3):e1009375. doi: 10.1371/journal.ppat.1009375 (PMC7984618; doi:10.1371/journal.ppat.1009375)

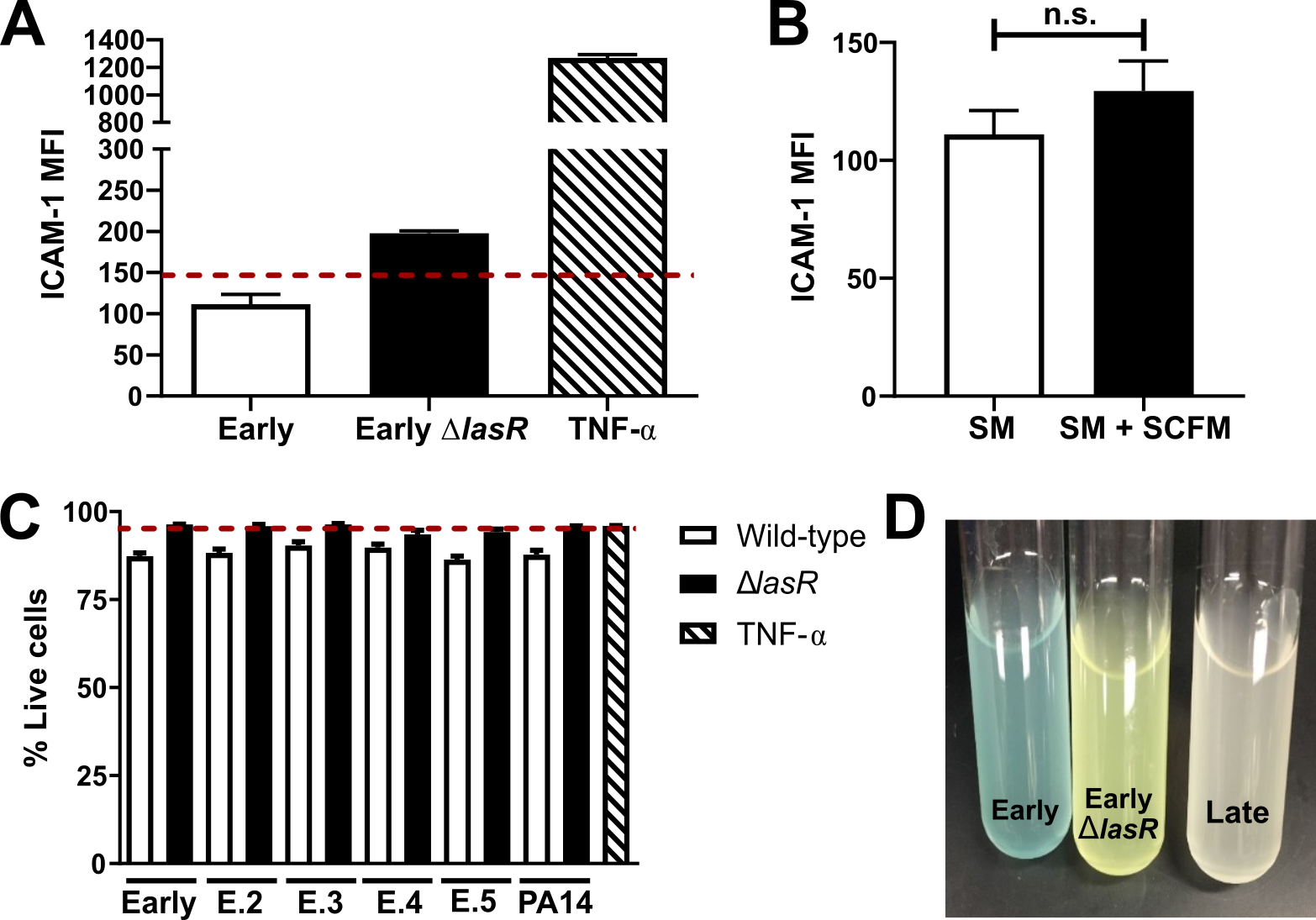

Supplement: S1 Fig — BEAS-2B cells were stimulated for 24h with (A) 30 μL filtrate of the Early or Early ΔlasR strain or 20 ng/mL TNF-α; (B) starvation media (SM) +/- 30 μL SCFM medium; (C) 30 μL filtrates from six pairs of wild-type clinical isolates and isogenic lasR mutant. In (A) and (C), SCFM served as negative control (—dashed line) and 20 ng/mL TNF-α as positive control. (A+B) mICAM-1 levels were measured by flow cytometry and (B) AEC viability following filtrate stimulation was measured as the percentage of live cells (low eFluor 780) among all single cells by flow cytometry. The results are shown as mean ± SD of one representative experiment (from ≥ 2 independent experiments, each with biological triplicates). (D) Representative bacterial cultures of the Early, Early ΔlasR and Late strains, with pyocyanin (blue-green pigment) production only evident with the Early strain. Cultures were grown in SCFM, as used for filtrate production. *P < 0.05; **P < 0.01; ***P < 0.001. (TIF) [file ppat.1009375.s001.tif]

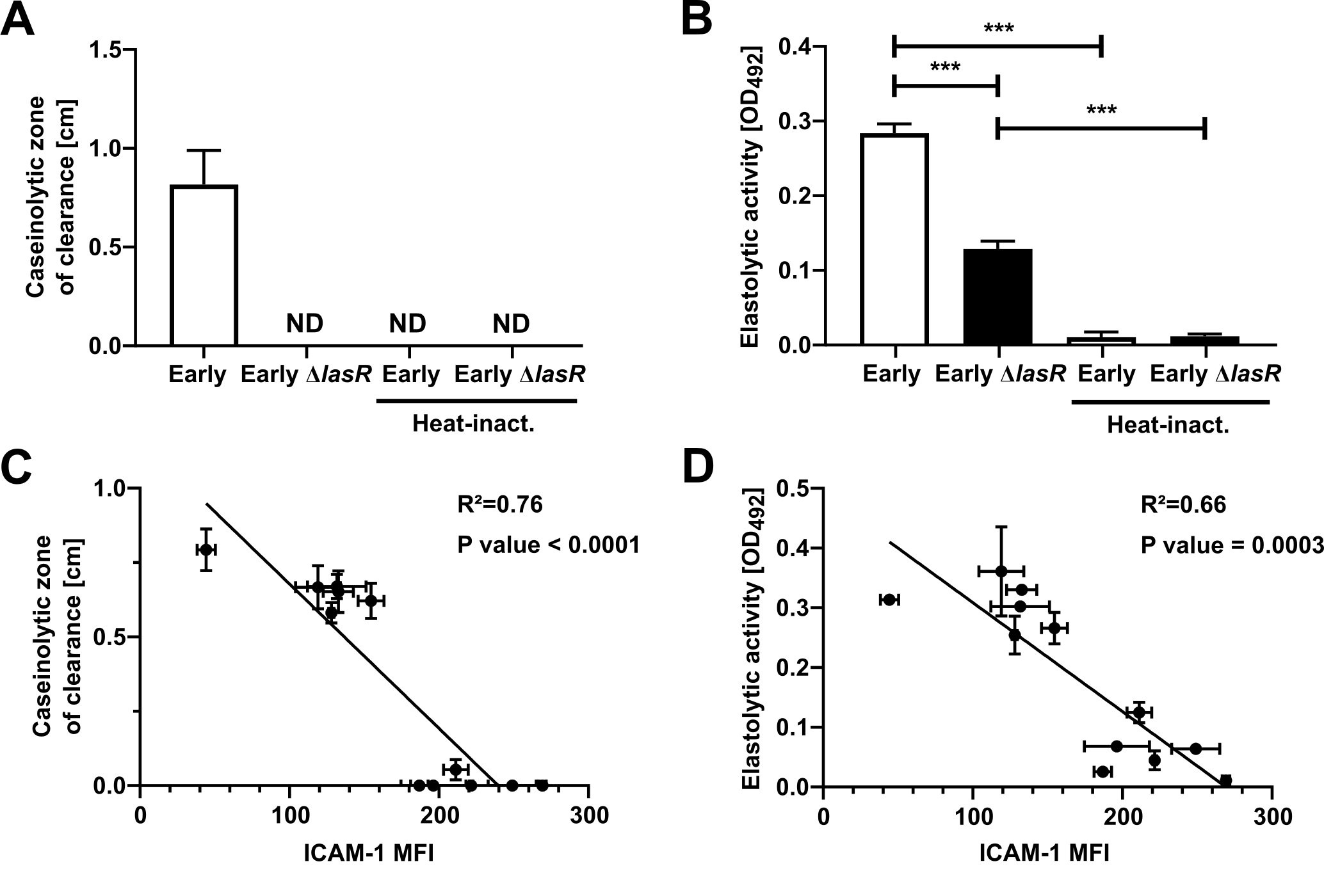

Supplement: S2 Fig — (A) Caseinolytic activity in Early and Early ΔlasR filtrates (+/- heat treatment) was measured on skim milk agar plates. (B) Elastolytic activity in Early and Early ΔlasR filtrates (+/- heat treatment) was measured by Elastin-Congo Red assay. Correlation between (C) caseinolytic or (D) elastolytic activity of different P. aeruginosa filtrates and mICAM-1 levels on AEC stimulated with the respective filtrates. Results in (A) and (B) are shown as mean +SEM and are representative of ≥ 2 independent experiments, each with biological duplicates. In (C) and (D), each data point represents one wild-type or lasR mutant strain, with the X value displaying the mean ±SD ICAM-1 induction in one representative experiment (in biological triplicates) and the Y value displaying the mean ± SEM (C) caseinolytic or (D) elastolytic activity (two independent experiments, each with biological duplicates). The trendlines in (C) and (D) were calculated by linear regression. ND = not detectable. *P < 0.05; **P < 0.01; ***P < 0.001. (TIF) [file ppat.1009375.s002.tif]

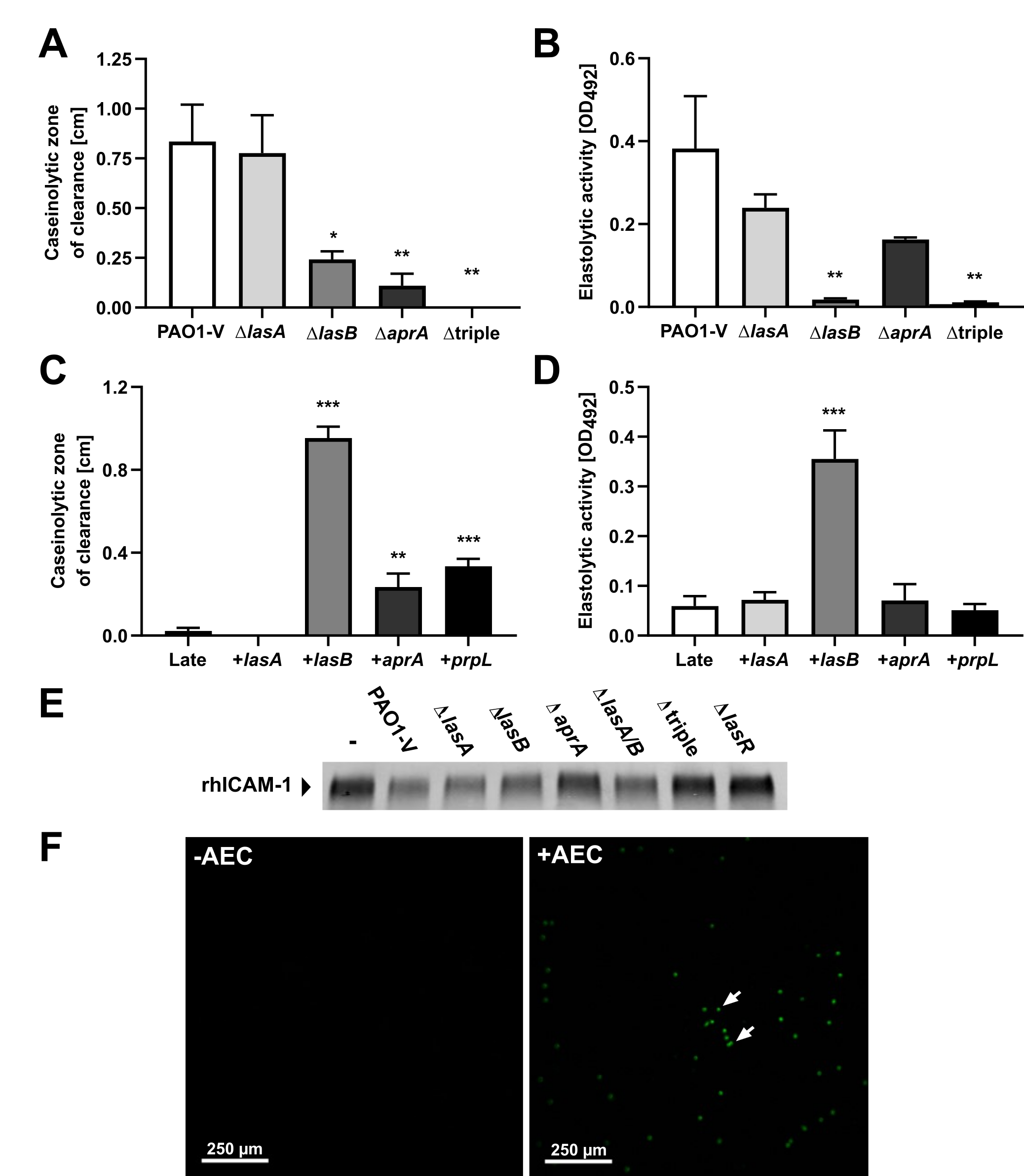

Supplement: S3 Fig — Caseinolytic (A+C) and elastolytic (B+D) activity of (A+B) PAO1-V and its isogenic protease mutants of lasA, lasB and aprA or (C+D) the Late strain complemented with lasA, lasB, aprA or prpL (T4P) was measured on skim milk agar plates and by Elastin-Congo Red assay, respectively. (E) rhICAM-1 was quantified by Western Blotting with a polyclonal anti rhICAM-1 antibody, following incubation for 24h with PBS (- control) or filtrates of PAO1-V and its isogenic protease mutants as indicated. (F) Adhesion of calcein-stained human primary neutrophils (green) after 2h of incubation in wells with or without AEC was analyzed by confocal imaging. Results in (A-D) are shown as mean ± SEM, with pooled data (n ≥ 3 biological replicates from ≥ 2 independent experiments). Results in (G) are representative of 2 independent experiments. Results in (F) are representative of 2 independent experiments. *P < 0.05; **P < 0.01; ***P < 0.001. (TIF) [file ppat.1009375.s003.tif]

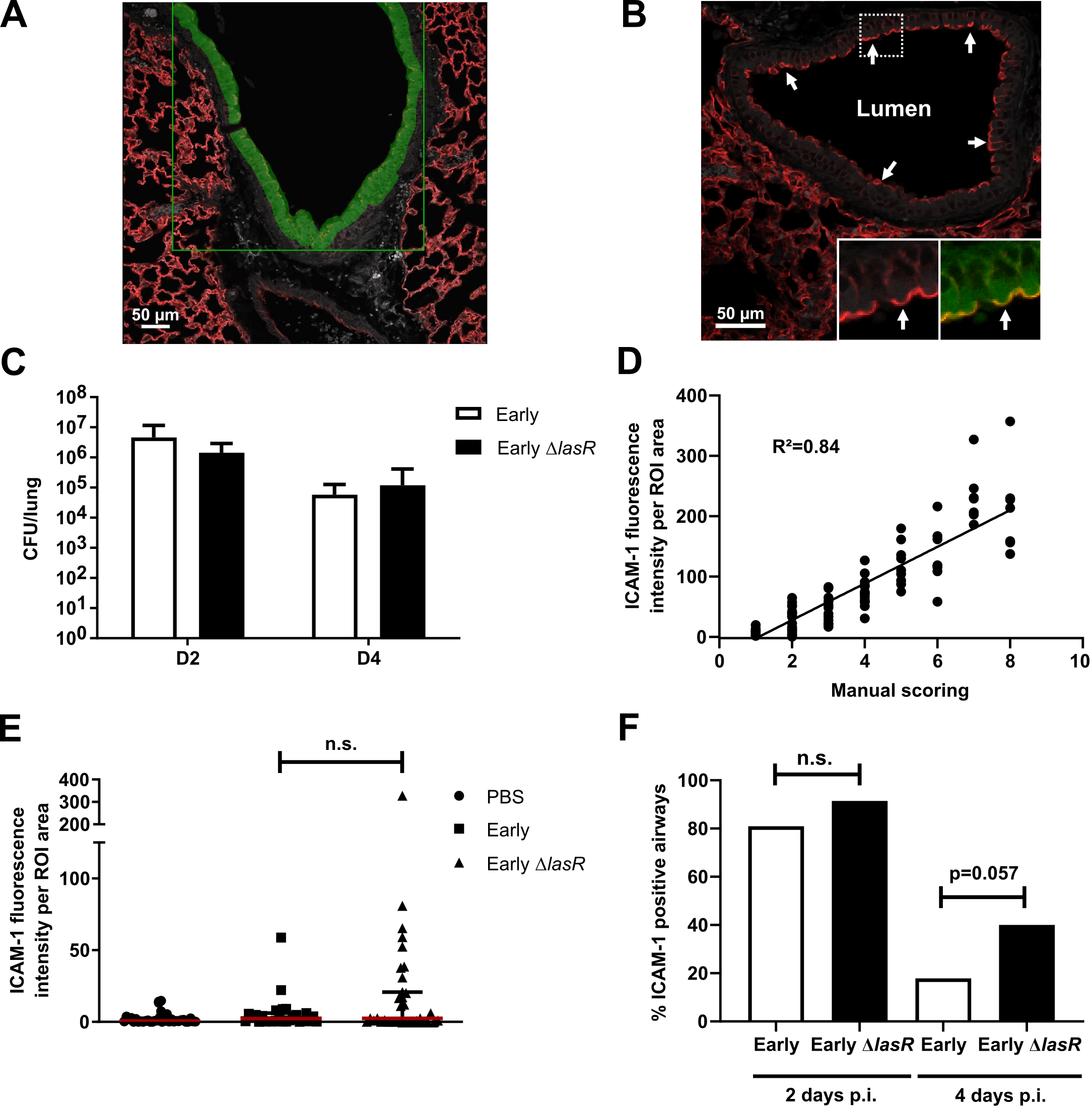

Supplement: S4 Fig — (A) Representative image of the region of interest (ROI, green area) manually drawn to define the airway epithelium on mouse lung sections. (B) Representative image of an airway cross-section (20X objective, with digital magnification in the inset box) displaying high bronchial ICAM-1 expression (red) localized to the apical side the bronchial epithelium facing the lumen (arrows). The autofluorescence of the tissue was imaged in the green channel (Ex 488/ Em 518) and is shown both in grey (to better highlight the ICAM-1 signal) or green. (C) Total lung bacterial burden at 2 and 4 days p.i, in mice infected with the Early or Early ΔlasR strain. Results are pooled from two independent experiments. (D) Correlation between manual and automated scoring of the airway epithelial mICAM-1 fluorescence intensity. Each dot represents one distinct airway section analyzed. (E) ICAM-1 fluorescence intensity per ROI area in mice infected with the Early, Early ΔlasR or PBS control. Each dot represents one airway section. (F) Percentage of ICAM-1 positive airways in mice infected with the Early or Early ΔlasR strain at 2 and 4 days p.i. Results are shown as mean ± SEM (C), median ± IQR (E) or percentages (F). *P < 0.05; **P < 0.01; ***P < 0.001; n.s. P ≥ 0.05. (TIF) [file ppat.1009375.s004.tif]

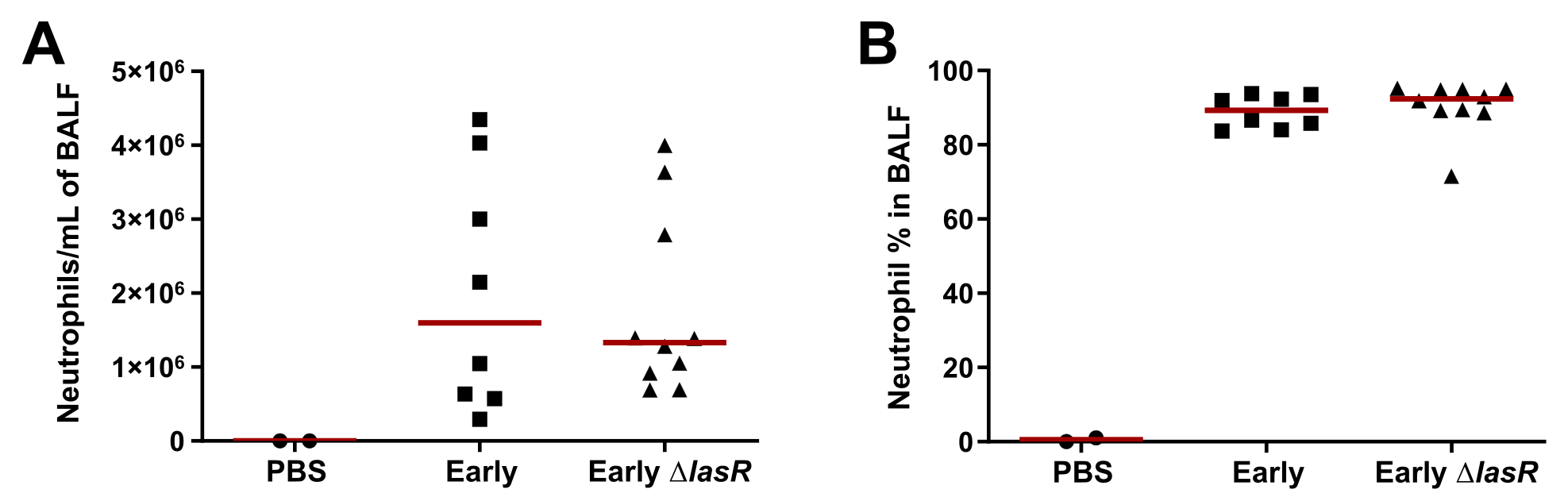

Supplement: S5 Fig — C57BL/6 mice were infected with the Early, Early ΔlasR or PBS control and sacrificed at 2 days p.i. (A) In BALF, the proportion of neutrophils was determined by Kwik-Diff staining. (B) Total BALF neutrophil counts, calculated by multiplying the proportion of neutrophils by the total number of live cells. Results are shown as medians (n = 2 mice in the control group, n ≥ 8 mice in infected groups). *P < 0.05; **P < 0.01; ***P < 0.001. (TIF) [file ppat.1009375.s005.tif]
