# Supplementary material for: LasR-deficient Pseudomonas aeruginosa variants increase airway epithelial mICAM-1 expression and enhance neutrophilic lung inflammation
Source: PLoS Pathog. 2021 Mar 10;17(3):e1009375. doi: 10.1371/journal.ppat.1009375 (PMC7984618; doi:10.1371/journal.ppat.1009375)
Supplement: S1 Table — (DOCX) [file ppat.1009375.s006.docx]

| **Strains** | **Genotype and relevant phenotypic characteristics** | **Reference** |
| --- | --- | --- |
| **Early** | “AMT0023-30” early infection clinical CF isolate with wild-type *lasR*  Additional information in Table S2 | [1] |
| **Early Δ*lasR*** | Early isolate with *lasR*::Gm^R^ | [2] |
| **Late** | “AMT0023-34” late infection clinical CF isolate clonally related to Early with loss-of-function *lasR* mutation  Additional information in Table S2 | [1] |
| **Late +*lasA*** | Late isolate with integrated attCTX::miniCTX2.1-Tc-GW-araC-pBAD::*lasA*, Tc^R^, resulting in arabinose-inducible *lasA* expression | This study |
| **Late +*lasB*** | Late isolate with integrated attCTX::miniCTX2.1-Tc-GW-araC-pBAD::*lasB*, Tc^R^, resulting in arabinose-inducible *lasB* expression | [3] |
| **Late +*aprA*** | Late isolate with integrated attCTX::miniCTX2.1-Tc-GW-araC-pBAD::*aprA*, Tc^R^, resulting in arabinose-inducible *aprA* expression | This study |
| **Late +*prpL*** | Late isolate with integrated attCTX::miniCTX2.1-Tc-GW-araC-pBAD::*prpL*, Tc^R^, resulting in arabinose-inducible *prpL* expression | This study |
| **E.2** | “CF215” early infection CF clinical isolate with wild-type *lasR* | [2] |
| **E.2 Δ*lasR*** | E.4 with *lasR*::Gm^R^ | [2] |
| **E.3** | “CF3-0.8” early infection CF clinical isolate with wild-type *lasR* | [2] |
| **E.3 Δ*lasR*** | E.5 with *lasR*::Gm^R^ | [2] |
| **E.4** | “CF6-1” early infection CF clinical isolate with wild-type *lasR* | [2] |
| **E.4 Δ*lasR*** | E.6 with *lasR*::Gm^R^ | [2] |
| **E.5** | “CF716” early infection CF clinical isolate with wild-type *lasR* | [2] |
| **E.5 Δ*lasR*** | E.7 with *lasR*::Gm^R^ | [2] |
| **E.6** | “c5198d” early infection clinical CF isolate with wild-type *lasR*. RAPD genotype A173. Additional information in Table S2 | This study |
| **L.6** | “D3010c” late infection clinical CF isolate clonally related to E.6 with loss-of-function *lasR* mutation. RAPD genotype A173. Additional information in Table S2 | This study |
| **E.7** | “AMT0020-1” early infection clinical CF isolate with wild-type *lasR*. Additional information in Table S2 | [3] |
| **L.7** | “AMT0020-84” Late infection clinical CF isolate clonally related to E.7. Additional information in Table S2 | [3] |
| **PA14** | Reference strain, isolated from a burn wound infection, wild-type *lasR* | [4] |
| **PA14 Δ*lasR*** | PA14 with *lasR*::Gm^R^ | [5] |
| **PAO1-V** | Invasive *P. aeruginosa* isolate with wild-type *lasR* | [6] |
| **PAO1-V Δ*lasA*** | PAO1-V with *lasA*ΩGm^R^ | [6,7] |
| **PAO1-V Δ*lasB*** | PAO1-V with Δ*lasB* deletion | [3] |
| **PAO1-V Δ*aprA*** | PAO1-V with Δ*aprA* deletion | [6,7] |
| **PAO1-V Δ*lasA* Δ*lasB*** | PAO1-V with *lasA*ΩGm^R^ and *lasB*ΩSm^R^ | [6,7] |
| **PAO1-V Δtriple** | PAO1-V with *lasA*ΩGm^R^, *lasB*ΩSm^R^, Δ*aprA* | [6,7] |
| **PAO1-V Δ*lasR*** | PAO1-V with *lasR*::Gm^R^ | [3] |
| ***E. coli* DH5α (pJN105L, pSC11)** | 3-oxo-C12-HSL bioassay strain | Ajai Dandekar (U Washington) |

**S1 Table. Strains used in this study.**

**References:**

1. Smith EE, Buckley DG, Wu Z, Saenphimmachak C, Hoffman LR, D'Argenio DA, et al. Genetic adaptation by Pseudomonas aeruginosa to the airways of cystic fibrosis patients. PNAS. 2006;103(22):8487-92.

2. D'Argenio DA, Wu M, Hoffman LR, Kulasekara HD, Déziel E, Smith EE, et al. Growth phenotypes of Pseudomonas aeruginosa lasR mutants adapted to the airways of cystic fibrosis patients. Molecular microbiology. 2007;64(2):512-33.

3. LaFayette SL, Houle D, Beaudoin T, Wojewodka G, Radzioch D, Hoffman LR, et al. Cystic fibrosis-adapted Pseudomonas aeruginosa quorum sensing lasR mutants cause hyperinflammatory responses. Science advances. 2015;1(6).

4. Rahme LG, Stevens EJ, Wolfort SF, Shao J, Tompkins RG, Ausubel FM. Common virulence factors for bacterial pathogenicity in plants and animals. Science. 1995;268(5219):1899-902.

5. Déziel E, Lépine F, Milot S, He J, Mindrinos MN, Tompkins RG, et al. Analysis of Pseudomonas aeruginosa 4-hydroxy-2-alkylquinolines (HAQs) reveals a role for 4-hydroxy-2-heptylquinoline in cell-to-cell communication. PNAS 2004;101(5):1339-44.

6. Hobden JA. Pseudomonas aeruginosa proteases and corneal virulence. DNA and cell biology. 2002;21(5-6):391-6.

7. Cowell BA, Twining SS, Hobden JA, Kwong MSF, Fleiszig SMJ. Mutation of lasA and lasB reduces Pseudomonas aeruginosa invasion of epithelial cells. Microbiology. 2003;149(Pt 8):2291-9.
