# Supplementary material for: LasR-deficient Pseudomonas aeruginosa variants increase airway epithelial mICAM-1 expression and enhance neutrophilic lung inflammation
Source: PLoS Pathog. 2021 Mar 10;17(3):e1009375. doi: 10.1371/journal.ppat.1009375 (PMC7984618; doi:10.1371/journal.ppat.1009375)
Supplement: S2 Table — (DOCX) [file ppat.1009375.s007.docx]

| Primer | Primer Sequence |
| --- | --- |
| *lasA*-GWB5-RBS | GGGGACAACTTTGTATACAAAAGTTGCCAGAGGAGGATATTCATGCAGCACAAAAGATCCCGC |
| *lasA*-GWB2 | GGGGACCACTTTGTACAAGAAAGCTGGGTATCAGAGCGCCAGGCCGGG |
| *aprA*-GWB5-RBS | GGGGACAACTTTGTATACAAAAGTTGCCAGAGGAGGATATTCATGTCCAGCAATTCTCTTG |
| *aprA*-GWB2 | GGGGACCACTTTGTACAAGAAAGCTGGGTATCAGACGACGATGTCGGCCT |
| *prpL*-GWB5-RBS | GGGGACAACTTTGTATACAAAAGTTGCCAGAGGAGGATATTCATGCATAAGAGAACGTACCTGAAT |
| *prpL*-GWB2 | GGGGACCACTTTGTACAAGAAAGCTGGGTATCAGGGCGCGAAGTAGCG |

**S2 Table. Primers used in this study.**
