# Supplementary material for: LasR-deficient Pseudomonas aeruginosa variants increase airway epithelial mICAM-1 expression and enhance neutrophilic lung inflammation
Source: PLoS Pathog. 2021 Mar 10;17(3):e1009375. doi: 10.1371/journal.ppat.1009375 (PMC7984618; doi:10.1371/journal.ppat.1009375)
Supplement: S3 Table — (DOCX) [file ppat.1009375.s008.docx]

| Plasmid | Description | Source or Reference |
| --- | --- | --- |
| pDONR221P5P2 | Multisite Gateway donor vector with attP5 and attP2 recombination sites, Cm^R^, Km^R^ | Invitrogen |
| pJJH187 | pDONR221P5P2 with an attL-flanked, 1192-bp fragment encoding the araC repressor and the pBAD promoter, Km^R^ | [1] |
| miniCTX2.1- GW | miniCTX2.1-Tc with a Gateway destination cloning site, Tc^R^ | J J Harrison |
| pENTR-*lasA* | pDONR221P5P2 containing the entire *lasA* ORF fragment (1257 bp), Cm^R^, Km^R^ | This Study |
| pENTR-*prpL* | pDONR221P5P2 containing the entire *prpL* ORF fragment (1389 bp), Cm^R^, Km^R^ | This Study |
| pENTR-*aprA* | pDONR221P5P2 containing the entire *aprA* ORF fragment (1483 bp), Cm^R^, Km^R^ | This Study |
| pEXP-*lasA* | miniCTX2.1-Tc-GW-araC-pBAD::*lasA*, Tc^R^ | This Study |
| pEXP-*prpL* | miniCTX2.1-Tc-GW-araC-pBAD::*prpL*, Tc^R^ | This Study |
| pEXP-*aprA* | miniCTX2.1-Tc-GW-araC-pBAD::*aprA*, Tc^R^ | This Study |

**S3 Table. Plasmids used in this study.**

**References:**

1. Khakimova M, Ahlgren HG, Harrison JJ, English AM, Nguyen D. The stringent response controls catalases in Pseudomonas aeruginosa and is required for hydrogen peroxide and antibiotic tolerance. Journal of bacteriology. 2013;195(9):2011-20.
