# Supplementary material for: LasR-deficient Pseudomonas aeruginosa variants increase airway epithelial mICAM-1 expression and enhance neutrophilic lung inflammation
Source: PLoS Pathog. 2021 Mar 10;17(3):e1009375. doi: 10.1371/journal.ppat.1009375 (PMC7984618; doi:10.1371/journal.ppat.1009375)
Supplement: S4 Table — Low protease production, the presence of a metallic sheen and low 3-oxo-C12-HSL signal level are characteristic phenotypes of LasR-deficient strains. NA not available. (DOCX) [file ppat.1009375.s009.docx]

| **Strains** | **Time interval** | ***lasR* genotype** | **Protease activity** | **Metallic sheen** | **3-oxo-C12-HSL level** |
| --- | --- | --- | --- | --- | --- |
| **Early** | - | Wild-type | Elastolytic ++  Caseinolytic ++ | Yes | ++ |
| **Late** | 7.5 years | 1 bp deletion at position 147 | Elastolytic +/-  Caseinolytic - | No | - |
| **E.6** | - | Wild-type | Elastolytic ++  Caseinolytic ++ | Yes | + |
| **L.6** | 15 years | T75K missense mutation | Elastolytic -  Caseinolytic - | No | - |
| **E.7** | - | Wild-type | Elastolytic ++  Caseinolytic ++ | Yes | ++ |
| **L.7** | 16 years | NA | Elastolytic -  Caseinolytic - | No | - |

**S4 Table. Characteristics of paired clonally related early and late infection isolates.** Low protease production, the presence of a metallic sheen and low 3-oxo-C12-HSL signal level are characteristic phenotypes of LasR-deficient strains. NA not available.
